# Quality of life and psychological wellbeing of adults with anaphylaxis: a mixed method systematic review

**DOI:** 10.1007/s11136-026-04266-0

**Published:** 2026-06-06

**Authors:** Bethany Arbuckle, Elizabeth Forster, Amy E. Mitchell

**Affiliations:** 1 Faculty of Health Sciences, School of Nursing, Midwifery and Paramedicine, Australian Catholic University, 1100 Nudgee Road, Banyo, QLD 4014 Australia; 2 School of Nursing and Midwifery, Griffith University, 170 Kessels Rd, Nathan, QLD 4111 Australia; 3 School of Nursing, Midwifery and Social Work, The University of Queensland, Sir Fred Schonell Drive, St Lucia, QLD 4072 Australia; 4 Parenting and Family Support Centre, School of Psychology, The University of Queensland, Sir Fred Schonell Drive, St Lucia, QLD 4072 Australia; 5 Griffith Centre for Mental Health, Griffith University, 176 Messines Ridge Road, Mt Gravatt, QLD 4122 Australia

**Keywords:** Systematic review, Anaphylaxis, Quality of life, Psychological well-being

## Abstract

**Purpose:**

To review the current evidence on quality of life (QOL) and psychological wellbeing of adults with anaphylaxis.

**Methods:**

A mixed method systematic review was conducted. The comprehensive search used the Preferred Reporting Items for Systematic Reviews and Meta-Analyses guidelines. Nine databases (MEDLINE, EBSCOhost, Embase, CINAHL Complete, Joanna Briggs Institute [JBI], Nursing and Allied Health ProQuest, PsycINFO, Cochrane Library and Google Scholar) were searched for literature published between January 2011 and October 2024. The Mixed Methods Appraisal Tool was used to assess methodological quality. Data from included studies were analysed using convergent mixed methods design. The protocol was prospectively registered (PROSPERO 2024 CRD42024583368).

**Results:**

A total of 11 papers (10 studies) met the inclusion criteria. Most studies reported that anaphylaxis has a negative impact on adults' psychological wellbeing, with many experiencing high levels of stress, anxiety, and/or depression. Women reported worse mental health than men. QOL was also affected, as most participants reported daily limitations, and many expressed reduced enjoyment in social activities. Impaired QOL was attributed to fear and emotional burden arising from previous episodes of anaphylaxis, contributing to avoidant behaviours and social withdrawal in an attempt to reduce the risk of future anaphylaxis events.

**Conclusion:**

Anaphylaxis affects adults’ psychological wellbeing and QOL. This review highlighted that clinicians may consider routine assessment of QOL and psychological wellbeing to not only provide adequate support but to identify patients who may require additional support as they learn to balance daily living with anaphylaxis.

**Supplementary Information:**

The online version contains supplementary material available at 10.1007/s11136-026-04266-0.

## Introduction

Anaphylaxis is increasing worldwide, with a global incidence estimated at 44 cases per 100,000 [1]. While prevalence is higher in children, adults experience greater mortality, and international data demonstrate rising incidence in food-related reactions; overall, 0.26% of hospital admissions globally are due to anaphylaxis, with the highest admission rates in Australia and the lowest in Spain, Taiwan and the United States [2–4]. While anaphylaxis is seen as an acute event, it is increasingly recognised as a chronic condition for some individuals, particularly those with recurrent anaphylaxis [5]. Given the potential for chronic health conditions to impact an individual’s psychological wellbeing and quality of life (QOL) [6], it is important to consider the psychological wellbeing and QOL of adults living with anaphylaxis as it can be a distressing and psychologically challenging experience.

Anaphylaxis is a medical emergency, defined by the World Health Organization [7] as a severe, life-threatening, or systemic hypersensitivity reaction that can progress from exposure to an allergen trigger. Those with a history of severe asthma are at the highest risk of anaphylaxis, and vitamin D insufficiency is linked to the increased likelihood of food-induced anaphylaxis [8]. Other risk factors for anaphylaxis include increased age, comorbidities, history of adverse drug reactions and allergies, mast cell disease, stress and social factors such as education and socioeconomic status [9].

Tang et al. [10] define psychological wellbeing as multidimensional and characterised by hedonic and eudaimonic aspects of mental health. It involves the experience of positive emotions such as pleasure, enjoyment, and life satisfaction (hedonia) as well as a deeper sense of purpose or meaning (eudaimonia). Psychological wellbeing also includes resilience, which encompasses coping strategies, and the ability to manage stress and regulate emotions effectively [10]. Carol Ryff’s [11] psychological wellbeing model depicts psychological wellbeing as being influenced by six different dimensions: including autonomy, environmental mastery, personal growth, positive relations with others, “purpose in life” and self-acceptance. When an individual's psychological wellbeing is compromised, they can experience issues with physical and psychological health, impacting the person's QOL [12].

The World Health Organization [13] defines QOL as an individual's perception of their position in life in the context of culture, values, and how they live in relation to their individual goals and expectations. QOL is a multidimensional construct encompassing physical health, psychological wellbeing, social relationships, environment, and functional status [14]. In individuals with anaphylaxis, ongoing risk management such as carrying rescue medication, avoiding potential triggers, and limiting social participation can affect daily functioning [15]. As with other chronic health conditions, when these adaptations are perceived negatively, they may impact mental health [15–17]. In the past, research exploring psychological wellbeing and QOL of adults with anaphylaxis has predominantly focused on adult parents of children with allergies, demonstrating increased levels of anxiety and depression compared to parents with children without allergies [18–21]. However, with the rising prevalence of adult-onset anaphylaxis, research is needed to understand the impact on QOL and psychological wellbeing. To date, there have been no systematic reviews examining the psychological wellbeing and quality of life among adults across all anaphylaxis triggers. While Martini et al. [22] reviewed drug hypersensitivity reactions, this focus does not capture the broader spectrum of anaphylaxis across all triggers. This gap highlights the need for comprehensive synthesis to inform evidence-based approaches when caring for this at-risk clinical group.

### Review aim

This review aimed to critically analyse and synthesise the current evidence relating to the QOL and psychological wellbeing of adults with anaphylaxis. The following research question underpinned the review: What is the QOL and psychological wellbeing of adults who experience anaphylaxis?

## Methods

### Protocol

The protocol was prospectively registered with PROSPERO (PROSPERO 2024 CRD42024583368).

### Eligibility criteria

The inclusion and exclusion criteria are summarised in Table 1. The review appraised research involving adults that were published from January 2011 to October 2024. This timeframe was selected to capture recently published literature following the development of the National Health Service (NHS) National Institute for Health and Care Excellence (NICE) Guideline on Anaphylaxis in 2011 [23]. Studies with mixed samples of participants that included children under the age of 18 and/or parents were excluded unless the data for adults with anaphylaxis were reported separately.

**Table 1:** Inclusion/exclusion criteria for studies

	Inclusion	Exclusion
General criteria	Published between 2011 and 2024 in peer-reviewed journals	Published before 2011 Not in a peer reviewed journal
Population	All adults with anaphylaxis, ≥ 18 years age	Adult parents of children with anaphylaxis, children under 18 years
Exposure	Anaphylaxis	Allergies only, no reported anaphylaxis
Outcomes	QOL and psychological wellbeing	Studies were excluded where the reporting of psychological wellbeing and/or quality of life outcomes was unclear, incomplete, or lacked sufficient detail to allow meaningful synthesis
Types of studies	Any study type and design of primary research (qualitative, quantitative, mixed methods)	Grey literature, summaries, commentaries, review documents, case studies

### Information sources and search strategy

An expert university health librarian was consulted to assist with development of the search strategy. A comprehensive search was performed using eight electronic databases: MEDLINE (via EBSCOhost), EBSCOhost, Embase, CINAHL Complete (via EBSCOhost), Joanna Briggs Institute (JBI) (via Ovid), Nursing and Allied Health ProQuest, PsycINFO (via Ovid) and Cochrane Library. The search terms included: (“QOL*” OR “Quality N3 Life*” OR “wellbeing” OR “well-being” OR “Health related QOL” OR “QOL*” OR “HRQOL*”) AND (“Anaphylaxis” OR “Allergic disease” OR “Allergic shock” OR “Severe allergic reaction” OR “Life threatening allerg*” OR “Allergic emergenc*” OR “Anaphylactic reaction*”) AND (“psychological wellbeing” OR “Psychological well-being” OR “Psychological stress” OR “Psychological resilience*” OR “Psychological state” OR “Psychological distress” OR psychological N3 (wellbeing or well being or well-being or wellness or stress* or resilience or state or distress)). To identify additional relevant research, a manual search was conducted via Google Scholar, and the references of eligible articles were searched for further potentially relevant publications.

### Study selection

After importing the search results into Covidence, the duplicates were removed. The titles and abstracts were then independently screened against the inclusion criteria by two authors (BA, EF), and ineligible articles were removed. Subsequently, the full texts of the remaining articles were independently reviewed by two authors (BA, EF) with any disagreements resolved by a third author (AEM).

### Data extraction and synthesis

Data extraction included key characteristics such as the author/s, year, country, study design, sample/setting, psychological wellbeing measures, QOL measures, anaphylaxis severity/characteristics, and the relevant findings. This review employed a convergent synthesis design, analysing qualitative and quantitative data separately but presenting the findings combined.

### Study quality appraisal assessment

The Mixed Methods Appraisal Tool (MMAT) version 2018 [24] was used to assess the quality of the study designs: qualitative and quantitative. The MMAT is a validated critical appraisal tool designed specifically for systematic reviews that include qualitative, quantitative, and mixed-method study designs, allowing a consistent assessment across methodologies. For each of the five methodological criteria, each study is reviewed and determined if the criteria are rated as “Yes”, “No” or “Can’t tell”. The tool does not utilise a numerical score; however, the appraisal focuses on key strengths and weaknesses across the domains such as sampling strategy, measurement validity, risk of bias, appropriateness of analysis, and data collection and interpretation. The MMAT was used in this review to inform the interpretation of findings and confidence in the evidence rather than as a basis for exclusion (see online Appendix 1).

## Results

### Search results

**Fig. 1:**
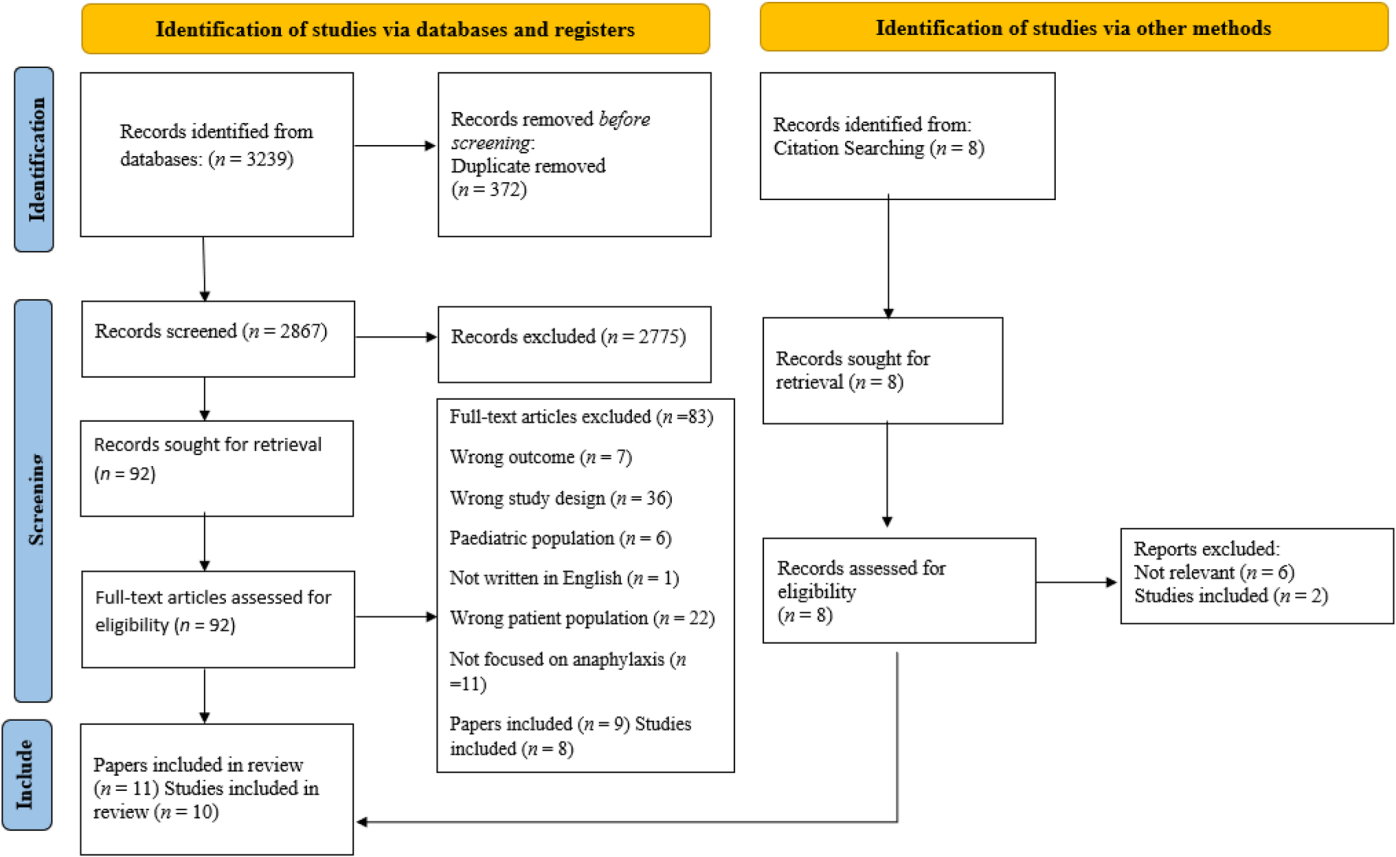
Quality of life and psychological wellbeing of adults with anaphylaxis: a mixed method systematic review

The database searches produced a total of 3239 records (see Fig. 1); after 372 duplicates were removed, and the remaining 2867 titles and abstracts were screened, 2775 were found to be ineligible [25]. The remaining 92 papers were screened through full-text review, of which nine papers reporting on eight studies were eligible for inclusion. A further eight studies were identified via hand searching references of all included studies, six were ineligible and two included within the full-text review, giving a total of 10 studies (11 papers) to be included in the review.

### Characteristics of the included studies

To ensure clarity in terminology, this review distinguishes between *studies* and *papers*. A total of 11 papers were included; however, these represented 10 unique studies, as two papers [15, 26] reported findings from the same underlying study population. For the purposes of this review, these papers were treated as a single study when describing study characteristics, while both papers are cited where relevant to their specific analyses. A detailed description of the study characteristics is provided in Table 2. Of the 10 included studies, eight used a quantitative cross-sectional study [15, 26–33] design and two used qualitative study designs [34, 35] with thematic analysis to gain insight into the participants' experiences. Within the review, 40% of the studies (k = 4) were published in the United Kingdom [15, 26, 29, 34, 35], with the remainder being published in Germany (k = 1) [31], Poland (k = 1) [33], the Netherlands (k = 1) [32], Denmark (k = 1) [28], Italy (k = 1) [30] or Turkey (k = 1) [27]. All studies included adult-only samples, except for two that compared subsamples of adults, adolescents, children, and parents of adolescents with anaphylaxis [27, 28].

**Table 2:** Study characteristics data extraction

Qualitative descriptive studies (k = 2)			
References(country)/design and methods	Sample and setting	Anaphylaxis measure/ allergy severity	Relevant findings
Knibb et al. [34] (United Kingdom) Qualitative semi-structured interviews thematic analysis	13 adults > 18 years with adult-onset anaphylaxis M = 56.8 years (40–71), 61% female, 100% White British, 38% retired, 53.8% employed, Clinic	World Allergy Organisation (WAO) diagnostic criteria. No severity is provided. 46% with Wasp allergy	Four key themes identified include fear of frustration, need to maintain a healthy identity, control over uncertainty, and the supportive role of others
Walklet et al. [35] (United Kingdom) Qualitative muti-perspective interviews thematic analysis	7 adults > 18 years with adult-onset anaphylaxis, M = 57.1 years (38–65), 3 family members, 2 Nursing staff, Ethnicity and SES not reported, 71% female, Clinic	57% with venom allergy	Two key themes identified: controllability and conflict. Control involved control over triggers and a lack of control causing distress Conflict covered the subtheme of rejecting identity

Cross-sectional studies (k = 8) papers (n = 9)				
References (country)	Sample, setting	Anaphylaxis severity and characteristics	Psychological (first) and QOL (Second) outcomes results/correlations	Other findings
Baiardini et al. [30] (Italy)	65 adults, M = 45.9 years, 80% female. Ethnicity and SES not reported Clinic	Episodes of anaphylaxis: 1 episode = 92% 2 episodes = 8% History of anaphylaxis and IgE mediated hypersensitivity to B-lactams	PGWBI M = 64.03 (SD = 17.66) (moderate distress): Positive wellbeing = 7.7%, No distress = 20.0%, Moderate distress = 32.3%, Severe distress = 40.0%. Total and domain scores significantly lower compared to population norms (*p* < 0.05)	Better DrHy-Q scores correlated with better PGWBI scores for anxiety, depressed mood, positive wellbeing, general health, and total score (r = − 0.25– − 0.41); no relationship with self-control or vitality
			DrHy-Q M = 62.82 (SD = 12.1) No correlation with age	
Chung et al. [29] (United Kingdom)	94 adults M = 44.2 years (18–76), 82% female. 100% White, 53% were from low income. Community-recruited via support organisation	Episodes of anaphylaxis: M = 3.9 (SD = 4.1). 73% anaphylaxis caused by food allergy	PCL: Intrusion M = 9.03 (SD = 4.06), Avoidance M = 11.79 (SD = 5.42), Arousal M = 8.67 (SD = 3.91), GHQ-28: 39% in clinical range, Somatic Sx M = 13.77 (SD = 5.65), Anxiety M = 13.37 (SD = 6.90), Social dysfunction M = 14.82 (SD = 2.90), Depression M = 9.36 (SD = 4.24) Reported feeling anxious about future anaphylaxis (75%), helpless to prevent future anaphylaxis (38%), angry about past anaphylaxis (33%)	PTS symptoms and psychiatric comorbidity predicted by characteristics of anaphylaxis experience, and predicted use of emotion-, avoidance- and problem-focused coping strategies
			No QOL outcomes	
Emre and Kan [27] (Türkiye)	163 (group 1 no anaphylaxis control group n = 71, group 2 adults with anaphylaxis n = 54, group 3 parents of adolescents with anaphylaxis n = 38 M = 36.5 years, 70.5% male. Ethnicity not listed nor SES. Clinic	Not utilised. No severity is provided. 42% of anaphylaxis caused by drug allergy	STAI-S scores: Group 1: 38.5 ± 10.2 STAI-T scores: Group 1: 43.2 ± 9.0. No correlation between age, education level, gender, and occupation	
			No QOL reported	
Knibb et al. [15] (United Kingdom) Same data set as Knibb et al. [26]	142 adults with anaphylaxis, M = 44.4 years (18–78), 59.9% female. 82.4% White, 32.4% with university degree as the highest level of qualification, Clinic The author confirmed that further data was collected in addition to [26] for the analysis	Brown grading: Mild/moderate = 33.8%, Severe = 64.1% Cause of anaphylaxis: Food: 30.2% General Anesthesia = 19% Spontaneous = 21.1% Wasp or bee venom = 16.9%	HADS: Anxiety M = 6.57 (SD = 4.81). Moderate-severe in 23.2% of men, 49.4% of women. Depression M = 3.82 (SD = 4.01) Moderate-severe in 16.1% of men, 12.4% of women. Anxiety and depression scores both higher than population norms for women (*p* < 0.01) but not men. PSS M = 23.42 (SD = 8.49), higher than population norm (*p* < 0.001)	Worse A-QOL-Adults scores correlated with worse anxiety (r = 0.69), depression (r = 0.54), and stress (r = 0.38) scores
			WHOQOL-BREF: Physical M = 15.15 (SD = 3.47), Psychological M = 14.81 (SD = 2.87), Social M = 15.26 (SD = 3.74), Environmental M = 15.78 (SD = 2.56) Social and environmental QOL is worse than population norm (*p* < 0.001); physical QOL better than population norm (*p* < 0.05). A-QOL-Adults: Total M = 2.18 (SD = 0.94). Emotional M = 2.54 (1.07), Social M = 1.86 (SD = 0.94), Limitations M = 2.24 (SD = 1.07). Worse A-QOL-Adults scores correlated with worse WHOQOL-Brief physical (r = – 0.50), psychological (r = – 0.45), social (r = – 0.36) and environmental (r = – 0.48) scores	
Knibb et al. [26] (United Kingdom) Same data set as Knibb et al. [15]	115 patients M = 42.7 years, SD = 16.85, (18–78), 60% female, 83.5% white, 32.7% with tertiary degree. Clinic	No. of reactions M = 3.86. Food allergy triggered anaphylaxis 37.4%. 28% had a family history of allergies. 51% had a history of other allergies	HADS scores were positively correlated with anxiety and depression in participants who had experienced spontaneous anaphylaxis, while PSS scores were varied across each allergen trigger with *p*-values venom allergy (0.34), food allergy (0.43), drug allergy (0.08) and spontaneous allergy (0.26)	Worse A-QOL-Adults total and subscale scores correlated with greater HADS anxiety and depression (r = 0.43 − .72) scores, and greater PSS (r = 0.34 − .49) scores
			Worse A-QOL-Adults total and subscale scores correlated with worse WHOQOL BREF total and subscale scores (r = − 0.23– − 0.51)	
Nowak et al. [33] (Poland)	61 patients, Allergic to wasp M = 43 years (IQR = 36–54), 54% male, hx anaphylaxis. Allergic to bee venom (M = 47, IQR = 39–61) were 54% female, with no ethnicity reported. Education varied, with 34% of the wasp group holding vocational qualifications and 42.5% of the bee venom group holding higher education. Occupationally, 37.1% of the wasp group worked in outdoor physical roles, while 38.5% of the bee venom group were unemployed. Clinic	57% wasp allergy, 42.6% bee allergy	HADS: Anxiety: Wasp M = 20, IQR = 10–69, Bee M = 15, IQR = 5–39.5	
			VQLQ: Wasp Venom = 4.45, IQR = 3.22–5.75, Bee Venom M = 5.29, IQR = 4.71–6.17. Those with wasp venom allergy had lower VQLQ scores compared to those with an allergy to bee venom indicating poorer QOL	
Schaarschmidt et al. [31] (Germany)	55 adults, M = 53.7 years SD = 12.1, (23–77), 54.5% female. Ethnicity and SES not Reported, University Medical Center	Anaphylaxis to insect venom. Bee venom 12.7%. Wasp Venom 81.8%. Mueller scale: Grade 1 = 10.9%, Grade 2 = 45.5%, Grade 3 = 36.4%, Grade 5 = 7.3%, Duration of disease 5.1 + = 6.1 years	HADS Anxiety: Normal range: 72.7%, Borderline: 12.7%, Clinical range: 14.5%. HADS Depression: Normal range: 85.5%, Borderline: 9.1%, Clinical range: 5.5%. No correlation with age, status of VIT, WTP per month, or willingness to carry emergency medication	Patients were willing to pay 1727 Euro to remove the condition
			No QOL reported	
Stensgaard et al. [28] (Denmark)	386 participants, > 18 years n = 29, m = 26.89 years, SD = 11.67, 68.9% female. 60% hx anaphylaxis. Ethnicity not listed nor SES. Hospital. Reported on adolescents and children, however, reported data separately for adults hence why included	Sampson's severity score for Anaphylaxis: Grade 1 = 0, Grade 2 = 0, Grade 3 = 24%, Grade 4 = 69%, Grade 5 = 7%. 62% peanut allergy. 62% nut allergy, 31% egg allergy, 21% fruits, 21% other. Time since anaphylaxis: Very recently 14%, 6–12 month 10%, 2 years 17% more than two years 17%	No Psychological wellbeing reported	No correlation between Samson’s rating scale and FAQAQ-AF scores
			FAQAQ-AF total scores: Men: M = 3.06 (SD = 1.40), Women: M = 4.05 (SD = 1.38) FAQAQ-AF subscale scores: Allergen avoidance and restriction: Males M = 3.30 (SD = 1.37), Females M = 4.0 (SD = 1.33), Emotional impact: Males M = 3.68 (SD = 1.29), Females M = 4.44 (SD = 1.36), Risk of accidental exposure: Male M = 3.03 (SD = 1.69), Female M = 4.34 (SD = 1.46). Food allergy-related health: Male M = 2.21(SD = 1.25), Females M = 3.43 (SD = 1.38). Females' patients reported greater impact of food allergy on HRQOL than males. The severity of allergic reactions did not contribute significantly to HRQOL	
Van der Velde et al. [32] (Netherlands)	N = 73 adults > 18 years M = 35.8 years,79% female, 85% hx anaphylaxis. Ethnicity not listed nor SES, Clinic. Reported adolescents and children, however, reported adults separately	Peanut allergy 27%, Nut allergy 16%, milk allergy 3%, Egg allergy 4%, Wheat allergy 11%, soy allergy 5%, sesame allergy 5%	No Psychological wellbeing reported	
			FAQLQ scores: 4.35	

Coping orientation to problems experienced inventory (COPE): higher scores on specific subscales indicate greater reliance on that coping strategy. Drug hypersensitivity quality of life questionnaire (DrHy-Q): Scored 15–75. Food allergy quality of life questionnaire – Adult Form (FAQLQ-AF): Each question is scored 1–7. Hospital Anxiety and Depression Scale (HADS): Scored 0–21. Perceived Stress Scale (PSS): Scored 0–40. Posttraumatic Stress Disorder Checklist (PCL): Scores range 0–85. Psychological General Well-Being Index (PGWBI): Scores range 0–110. State-Trait Anxiety Inventory (STAI): Scores range 20–80. Vespid Allergy Quality of Life Questionnaire (VQLQ): Each question is scored 1–7. World Health Organization Quality of Life-Brief (WHOQOL-BREF): Scores range 0–100. Anaphylaxis Quality of Life Scale for Adults (A-QOL-A): Scores range from 1–5

The quantitative studies included participants with venom allergies (k = 2) [31, 33], food allergies (k = 3) [28, 29, 32], or drug allergies (k = 2) [27, 30], and one study (k = 1) [15, 26] focused on all three triggers. Both qualitative studies (k = 2) included participants with mostly venom allergies [34, 35]. The studies were conducted either in allergy clinics (k = 7) [15, 26, 27, 30, 32–35] or hospital outpatient (k = 1) [28] departments, although two (k = 2) used community recruitment [29, 31]. The severity of anaphylaxis varied across the studies, with several grading systems utilised such as the Mueller’s classifications (k = 1) [31], Brown’s grading (k = 1) [15, 26] and Sampson’s severity score (k = 1) [28]. Only three studies reported ethnicity, all from the United Kingdom and all reporting White samples [15, 26, 29, 34]. Indicators of socioeconomic status (SES) were reported in four studies [15, 26, 29, 33, 34]. Chung et al. [29] reported that 53% of their participants were from low socioeconomic backgrounds. The study conducted by Knibb et al. [26] reported an average of 32.7% of participants held a university degree as their highest level of education. Within the studies, there was variability in age and allergy type; however, eight out of the 10 studies had more female than male participants [15, 26, 28–32, 34, 35].

### Quality assessment

The MMAT was used for quality appraisal of the included studies [24] (see online Appendix 1). In six of the 10 studies, at least one criterion was not met [26, 28–32]. However, due to the limited literature on the topic, these studies were included. With the quantitative descriptive studies (k = 8), most did not meet the criteria for quality appraisal (k = 6) due to insufficient reporting within the studies [26, 28–32]. This assessment included an evaluation of whether the sampling strategy was appropriate to address the research question. This information was not reported in two studies [29, 31]. In four studies, insufficient detail was provided regarding sample characteristics and population representativeness [26, 28, 30, 32]. In six studies, the risk of bias was unclear due to inadequate reporting [26, 28–32]. All quantitative studies reported appropriate measurement procedures, including evidence of validity, and employed statistical analyses that were suitable for addressing the research question [15, 26–33]. Across all included quantitative and qualitative studies, the research questions were clearly stated, and data collection methods were adequate to address these questions [15, 26–35]. For the quantitative studies, response rates to surveys varied from 76 to 94%. Therefore, according to Elston [36], the risk of nonresponse bias is considered low as the response rate is over 60%. All MMAT criteria were met in the qualitative studies [34, 35] (see online Appendix 1).

### Quality of life-quantitative findings

Overall, five quantitative studies [15, 26, 28, 30, 32, 33] examined QOL outcomes. A summary of the results is presented in Table 2. The studies utilised four different self-report scales to measure QOL. Most studies utlised QOL measures specific to the allergy type such as food (k = 2) [28, 32], drug (k = 1) [30] or vespid allergy (k = 1) [33]. One study utilised the Anaphylaxis Quality of Life Scale for Adults (A-QoL-Adults), a measure designed to assess quality of life across multiple allergen triggers [15, 26]. In contrast to allergen-specific instruments, the A-QoL-Adults enables comparison of quality of life across different causes of anaphylaxis. For further information on specific measures, see online Appendix 2.

Knibb et al. [26] developed and validated the A-QoL-Adults, demonstrating its association with general quality of life measures. Subsequently, Knibb et al. [15] applied the A-QoL-Adults in adults with anaphylaxis and found that poorer scores were significantly correlated with lower WHOQOL-BREF domain scores, including physical (r =  − 0.50), psychological (r =  − 0.45), social (r =  − 0.36), and environmental (r =  − 0.48) quality of life. Importantly, the use of the A-QoL-Adults allowed for comparison across allergen triggers. Knibb et al. [15] reported that venom allergy was associated with poorer overall quality of life, while food allergy was linked to reduced psychological and environmental quality of life; no significant associations were identified for drug-induced anaphylaxis.

One study that used a general measure of QOL (WHOQOL-BREF) found reduced QOL for adults with anaphylaxis compared to population norms, with lower scores in the social and environmental QOL domains [26]. Two studies using the FAQLQ-AF [28, 32] reported consistently poorer quality of life among adults with anaphylaxis, with greater impairment observed in younger adults, women, and individuals with more frequent or severe episodes. One study assessed drug hypersensitivity-related QOL (DrHy-Q) [30] and found adults with drug-induced anaphylaxis had higher scores (poorer QOL) when compared to those who had not experienced an anaphylactic reaction. The one study that assessed QOL related to vespid allergy (VQLQ) [33] found poorer QOL among those with wasp venom allergy compared to those with bee venom allergy. As these QOL tools are allergen-specific and measure QOL within specific trigger contexts, direct numerical comparison of QOL scores across these allergens specific measures were not possible. Therefore, cross-allergen comparisons were interpreted through examining patterns of findings across studies. In summary, the included studies tended to report poorer overall QOL for individuals with anaphylaxis compared to population norms, with particularly poor QOL among women, younger adults, those with food allergy, and those experiencing recurrent anaphylactic episodes [26, 28, 32].

### Psychological wellbeing- quantitative findings

Across the quantitative studies, psychological wellbeing was consistently poorer than population norms. Overall, six quantitative studies [15, 26, 27, 29–31, 33] examined psychological outcomes. Participants reported elevated anxiety, depression and stress when assessed using validated measures such as the Hospital Anxiety and Depression Scale (HADS) (k = 3) [15, 26, 31, 33], the Perceived Stress Scale (PSS) (k = 1) [15, 26], the State-Trait Anxiety Inventory (STAI) (k = 1) [27] and the Posttraumatic Stress Score in the General Health Questionnaire (GHQ) (k = 1) [29].

Baiardini et al. [30] specifically measured overall psychological wellbeing using the Psychological General Well-Being Index (PGWBI) which is the only psychological wellbeing scale that assesses positive wellbeing, and reported findings that 72.3% of participants experienced moderate to severe distress, with mean PGWBI scores (M = 64.03, SD = 17.66) significantly lower than population norms (p < 0.05). Similarly, Knibb et al. [15] reported higher-than-average anxiety (M = 6.57, SD = 4.81) and stress (M = 23.42, SD = 8.49), with women showing greater rates of anxiety and depression than men using the Hospital Anxiety and Depression Scale (HADS). Knibb et al. [26] found that increased anxiety, depression, and perceived stress were strongly correlated with poorer quality of life (r = 0.34–.72). Chung et al. [29] reported that 39% of participants scored within the clinical range on the GHQ-28, alongside persistent fear and helplessness. Additionally, post-traumatic symptoms and psychiatric comorbidity were associated with increased use of emotion-focused, avoidance-focused, and problem-focused coping strategies on the COPE inventory; however, mean COPE subscale scores were not reported. The study revealed that people with anaphylaxis relied heavily on acceptance focused and problem-solving coping strategies with 93% accepting their illness. Emre and Kan [27] observed elevated state and trait anxiety among adults with anaphylaxis compared to controls, though anxiety did not correlate with demographic variables.

The studies that reviewed venom allergies showed variable outcomes. For instance, Nowak et al. [33] reported higher anxiety among those allergic to wasp venom versus bee venom, while Schaarschmidt et al. [31] found 14.5% of participants in the clinical range for anxiety and 5.5% for depression. Schaarschmidt et al. [31] reported that, on average, adults with anaphylaxis were willing to pay substantial amounts up to €1,727 to eliminate their allergies, highlighting the perceived burden of the condition. These findings indicate that adults with anaphylaxis experience significant psychological distress, characterised by increased anxiety, stress, coping strategies that persist beyond the acute episode.

### Qualitative study findings

Two qualitative studies (k = 2) explored the experience of adults with anaphylaxis [34, 35]. Walklet et al. [35] reviewed psychological wellbeing and QOL and identified two overarching themes of controllability and conflict highlighting individuals' struggles with illness identity. Feelings of fear were reported in both studies by participants to increase anxiety [34, 35]. Knibb et al. [34] reviewed QOL and reported that adults with anaphylaxis perceived the illness as “bad luck” affecting QOL due to constant hypervigilance, social avoidance, and fear of recurrence. Social avoidance was a recurring theme with participants disclosing withdrawal or avoidance at events, dining out and travel due to the fear of exposure to their allergen. Both studies identified anaphylaxis related psychological burden to be participants' feelings of perceived lack of control [34, 35].

## Discussion

This review aimed to critically analyse and synthesise current evidence relating to the QOL and psychological wellbeing of adults with anaphylaxis. Overall, the findings demonstrate that adults living with anaphylaxis experience significantly higher levels of stress, anxiety, and depression compared with individuals without anaphylaxis. Psychological distress in this population appears to be strongly influenced by anticipatory factors, particularly fear of future anaphylactic reactions and the ongoing need for vigilance and adopting avoidant behaviours. These findings align with previous systematic reviews examining drug hypersensitivity reactions, which similarly report substantial psychological burden and impaired QOL among adults with drug-induced anaphylaxis [22]. In addition, a review focusing on self-management interventions for psychological wellbeing in individuals with allergic conditions, including anaphylaxis, reported negative impacts on both QOL and psychological wellbeing [37]. While these reviews establish the psychosocial consequences of anaphylaxis, the present review extends existing literature by identifying specific contributors to poorer psychological outcomes, particularly fear of recurrence and avoidant behaviours, which have not been comprehensively explored in earlier syntheses. Consequently, clinicians may need to consider these contributors when assessing risk and planning care for adults with anaphylaxis.

Fear of future allergic reactions and its impact on daily living may vary depending on the allergen trigger however; this review demonstrates that reduced QOL is reported across all allergen types. Within Ferrans’ Revised Health-Related Quality of Life framework (2005) [38], this reflects the way perceived health threats influence psychological wellbeing and social functioning regardless of the source of risk. This finding is consistent with qualitative work by Roleston et al. [39], who explored the psychological burden of food allergy in adults and identified themes of pervasive fear and uncertainty, including fear of death following accidental exposure and reliance on avoidance strategies. Such fear-driven behaviours align with the general health perception and quality of life domains of Ferrans’ model [38], whereby ongoing vigilance and uncertainty disrupt daily functioning and participation. Notably, avoidant behaviours were particularly prominent among the participants diagnosed in childhood, suggesting long-term psychological adaptation to perceived risk [39]. Similarly, our findings indicate that fear of recurrence and avoidant behaviours are prevalent regardless of allergen trigger, with consistent negative effects on QOL. From a theoretical perspective, this suggests that the subjective experience of threat, rather than allergen type, is a primary driver of QOL impairment. Consequently, clinicians should not assume that non-food allergens confer lower psychosocial burden and may consider routine assessment of QOL and psychological wellbeing across all adults with anaphylaxis.

Comparisons with conditions that predispose individuals to anaphylaxis, such as mastocytosis, further contextualise these findings. Mastocytosis is a rare disorder characterised by mast cell activation and frequent anaphylactic reactions [40], placing affected individuals at heightened risk of psychological distress. Schmidt et al. [40] reported significantly lower global health scores among individuals with systemic mastocytosis and mast cell activation syndrome compared with healthy controls. Similarly, Spolak-Bobryk et al. [41] found that 27% of adults with mastocytosis experienced anxiety, 12.9% reported depression, and 15.3% reported low life satisfaction. These parallels suggest that recurrent or unpredictable anaphylactic risk, regardless of underlying diagnosis, is strongly associated with impaired QOL and psychological wellbeing. A review by Correia et al. [42] suggests elements that may support psychological wellbeing in those with chronic disorders include psychological support, education, and introducing coping strategies.

The psychological challenges identified in adults with anaphylaxis are not unique to this condition and mirror those observed in other chronic illnesses requiring ongoing self-management. Individuals with asthma and allergies frequently engage in environmental avoidance to prevent symptom exacerbation [43], while those with chronic obstructive pulmonary disease (COPD) often avoid physical and social activities due to fear of breathlessness [44]. Similarly, adults with anaphylaxis report avoidance of specific environments and social situations to minimise perceived risk. While such behaviours may be initially protective, prolonged, or excessive avoidance can become maladaptive, contributing to social isolation, heightened anxiety, and further reductions in QOL [45]. Clinical care approaches may need to consider addressing adaptive coping, exposure planning, and resilience-building to prevent maladaptive avoidance and optimise long-term psychological wellbeing.

## Implications

The findings of this review highlight the importance of addressing quality of life and psychological wellbeing as integral components of anaphylaxis care. Routine assessment of QOL and psychological wellbeing should be considered as part of ongoing care for adults with anaphylaxis, as fear of future reactions, vigilance, and avoidance behaviours were consistently associated with impaired QOL across allergen triggers. These implications reflect the consistent patterns identified across the included studies, despite variability in study design and quality, reinforcing the clinical relevance of psychological wellbeing assessment alongside quality of life. Clinicians should not assume that psychosocial burden is confined to specific allergen types, and consideration of QOL and psychological wellbeing may support more tailored, patient-centred care approaches for adults managing ongoing anaphylactic risk. From a research perspective, the findings underscore the importance of strengthening methodological approaches in future studies, including the use of representative samples, comprehensive reporting of participant characteristics, and the selection of multidimensional QOL and psychological wellbeing measures aligned with theoretical frameworks. Adopting such approaches may enhance the clinical applicability of future evidence and support the development of care strategies that address both physical risk and long-term psychosocial outcomes.

Collectively, these implications support a holistic, biopsychosocial care approach to anaphylaxis that extends beyond acute reaction management to include routine assessment and support of psychological wellbeing, quality of life, thereby improving long-term outcomes for adults living with anaphylaxis.

## Limitations

Several limitations should be considered when interpreting the findings of this review. Small convenience-based samples may not be representative of the broader adult population and could introduce sampling bias. The majority of studies were conducted in Europe, limiting inclusion of participants from other geographic locations. Variability in study design, measures of psychological wellbeing and QOL, and the allergen triggers assessed limited comparability across studies. Despite these limitations, the review synthesises the best available evidence on psychological wellbeing and QOL among adults with anaphylaxis, highlighting patterns of fear, avoidance, and reduced quality of life among adults with anaphylaxis.

## Conclusion

Overall, the findings of this review suggest that fear of future reactions and avoidant behaviours are central drivers of impaired QOL and psychological wellbeing in adults with anaphylaxis. Anaphylaxis has a negative impact on adults' psychological wellbeing, with many experiencing high levels of stress, anxiety, and depression. Negative impacts on the QOL were greater among females and correlated with fear and emotional burden often leading to adopting avoidance behaviours and social withdrawal. Assessment of QOL and psychological wellbeing should be considered as part of routine care for adults with anaphylaxis to enable provision of appropriate support for psychological wellbeing and QOL.

## Supplementary Information

Below is the link to the electronic supplementary material.

Supplementary Material 1

## Data Availability

All data supporting the findings of this study are available within the paper.
