# Supplementary material for: Quality of life and psychological wellbeing of adults with anaphylaxis: a mixed method systematic review
Source: Qual Life Res. 2026 Jun 6;35(7):183. doi: 10.1007/s11136-026-04266-0 (PMC13242375; doi:10.1007/s11136-026-04266-0)
Supplement: Supplementary file 1 — Supplementary Material 1 [file 11136_2026_4266_MOESM1_ESM.docx]

**Appendices**

#### Appendix 1

Mixed Method Appraisal Tool Results [25]

|  | **SCREENING QUESTIONS** | | **1. QUALITATIVE STUDIES** | | | | |
| --- | --- | --- | --- | --- | --- | --- | --- |
|  | S1. Are there clear research questions? | S2. Do the collected data allow to address the research questions? | 1.1. Is the qualitative approach appropriate to answer the research question? | 1.2. Are the qualitative data collection methods adequate to address the research question? | 1.3. Are the findings adequately derived from the data? | 1.4. Is the interpretation of results sufficiently substantiated by data? | 1.5. Is there a coherence between qualitative data sources, collection, analysis, and interpretation? |
| Knibb et al. 2019 [35] | √ | √ | √ | √ | √ | √ | √ |
| Walker et al. 2018 [36] | √ | √ | √ | √ | √ | √ | √ |

**SCREENING QUESTIONS 4****. QUANTITATIVE DESCRIPTIVE STUDIES**

|  | S1. Are there any clear research questions? | S2. Do the collected data allow to address the research questions? | 4.1. Is the sampling strategy relevant to address the research question? | 4.2. Is the sample representative of the target population? | 4.3. Are the measurements appropriate? | 4.4. Is the risk of nonresponse bias low? | 4.5. Is the statistical analysis appropriate to answer the research question? |
| --- | --- | --- | --- | --- | --- | --- | --- |
| Chung et al. 2011 [29] | √ | √ | X | √ | √ | X | √ |
| Schaarschmidt et al. 2018 [32] | √ | √ | X | √ | √ | X | √ |
| Baiardini et al. 2025 [31] | √ | √ | √ | X | √ | X | √ |
| Knibb et al. 2022 [30] | √ | √ | √ | X | √ | X | √ |
| Knibb et al. 2023 [16] | √ | √ | √ | X | √ | X | √ |
| Nowak et al. 2015 [34] | √ | √ | √ | √ | √ | √ | √ |
| Emre & Kan, 2022 [27] | √ | √ | √ | √ | √ | √ | √ |
| Van der Velde et al. 2012 [33] | √ | √ | √ | X | √ | X | √ |
| Stensgarrd et al. 2017 [28] | √ | √ | √ | X | √ | X | √ |
| Key:  √= Met criteria  X= Unmet or unclear |  |  |  |  |  |  |  |

#### Appendix 2

*Quality of Life and Psychological Wellbeing Scales*

| QOL Scales |  |  |  |  |  |
| --- | --- | --- | --- | --- | --- |
| Scale | Definition and domains | Year Created | Scoring Interpretation | Validation | Reported in studies |
| World Health Organization QOL-BREF (WHOQOL-BREF) | Assesses QOL across four domains: physical health, psychological health, social relationships, and environment. This is a reliable questionnaire that assesses QOL across four domains with 26 items: physical health (7 items), psychological health (6 items), social relationships (3 items), and environment (8 items) [14]. | 1998 | Scores range from 0 to 100 per domain; higher scores indicate a higher QOL. | A study conducted by Kalfoss et al. [47] revealed that the tool was a reliable measure for all domains apart from the social domain which found marginal reliability | (k = 2) |
| Drug Hypersensitivity QOL Questionnaire (DrHy-Q) | Evaluates the impact of drug hypersensitivity reactions on patients' QOL. The scale for each question is on Likert scale of 1-7 and contains 15 items | 2011 | Scored between 15 to 75. Higher scores reflect greater QOL impairment. | The scale has been validated by several studies and translated into other languages [48]. | (k = 1) |
| Food Allergy QOL Questionnaire – Adult Form (FAQLQ-AF) | Measures the impact of food allergies on adults' QOL across various domains. The FAQLQ-AF is a validated tool that measures the impact of food allergies on adults' QOL across various domains and contains 29 items [50]. The domains include allergen avoidance and dietary restriction, emotional impact, risk of accidental exposure, and food allergy related health. Each question is on a Likert scale of 1-7 (1 not troubled at all to 7 extremely troubled), and a higher score signifies a greater QOL. | 2009 | Each question is on a scale of 1-7. Higher scores signify greater QOL impairment. | The FAQLQ-AF has been validated through several studies and data from Goossen et al. [49] concluded that creating an online version of the tool had a good construct validity and internal consistency while being able to discriminative for anaphylaxis vs no anaphylaxis. | (k = 4) |
| Vespid Allergy QOL Questionnaire (VQLQ) | Assesses QOL in individuals with vespid venom allergy. The tool contains 14 core items that are rated on a scale from 1-7. | 2002 | Each question is on a scale of 1-7. Higher scores denote a more significant QOL impairment. | A study that has validated this tool was the study that was conducted by Sin et al.[51] and revealed a coefficient (Cronbach a) of 0.97. | (k =1) |
| Anaphylaxis Quality of life Scale for Adults (A-QOL-Adults) | Assesses condition-specific quality of life measure for adults with anaphylaxis, designed to assess QOL regardless of allergen trigger. | 2022 | Each question is scale of 1-7. Higher scores indicate worse quality of life. | Developed and validated by Knibb et al. (2022) [30], demonstrated strong psychometric properties, including good construct validity and excellent internal consistency (Cronbach’s α) of 0.96. | (k = 1) |

| Psychological Wellbeing Scales |  |  |  |  |  |
| --- | --- | --- | --- | --- | --- |
| Scale | Definition | Year Created | Scoring Interpretation | Validation | Reported in studies |
| Hospital Anxiety and Depression Scale (HADS) | Assesses self-reported anxiety and depression levels. The depression subsection consists of 14 items and 7 items for anxiety. | 1983 | Scores: 0–7 (normal), 8–10 (borderline-Mild), 11–14 (abnormal- Moderate), 15-21 (Severe) | The HADS score has been widely validated when compared to the General Health Questionnaire [52]. | (k = 7) |
| Perceived Stress Scale (PSS) | Measures the perception of stress, focusing on feelings and thoughts during the last month. It is a 14-item scale that assesses the perception of stress over the period of a month [53]. | 1983 | Scored from 0-40. Scores: 0–13 (low stress), 14–26 (moderate stress), 27–40 (high stress). | The tool has been validated through the following review however it should be noted that the cohort studies were mostly in college students or workers [54]. | (k = 2) |
| Posttraumatic Stress Disorder Checklist (PCL) | A self-report measure assessing the symptoms of PTSD. This scale measures four subscales including somatic symptoms, anxiety and insomnia, social dysfunction and severe depression and has 28 items that are scored on a Likert scale. | 1993 | Scores range from 0 to 85; higher scores indicate greater PTSD symptom severity. | Internal consistency of the PCL-5 was high (alpha = .85), and the instrument showed an adequate convergent validity [55]. | (k = 1) |
| Coping Orientation to Problems Experienced (COPE) Inventory | Assesses a broad range of effective and ineffective ways to cope with a stressful life event. The scale comprises of 60 items that are self-reported and measures 15 distinct coping strategies. | 1989 | Higher scores on specific subscales indicate that an individual relies more heavily on those particular coping strategies. | Validated to conclude a Cronbach’s a of between 0.62 to 0.92 [56]. | (k = 1) |
| Psychological General Well-Being Index (PGWBI) | Evaluates self-representations of subscales including depressed mood, positive well-being, self-control and vitality. The PGWBI is a scale that assesses six affective through 28 items including anxiety, depressed mood, positive well-being, self-control, general health, and vitality. | 1960s | Scores range from 0 to 110; higher scores indicate better well-being while scores that are lower may suggest psychological distress. | A study conducted by Darves-Bornoz et al. [57] stated the GHQ-28 reliability and validity in screening for PTSD was a Cronbach's α coefficient (0.95). | (k = 1) |
| State-Trait Anxiety Inventory (STAI) | Differentiates between the temporary condition of "state anxiety" and the long-standing quality of "trait anxiety" and consists of a 20-item self-reported measure [58]. | 1970 | Scores range from 20 to 80; higher scores on both scales indicate greater anxiety. | In a Danish validation study conducted by Gustafson et al. [59] the reported Cronbach’s alpha of 0.93 confirmed high validity. | (k = 1) |

#### Appendix 3

Search strategy

EBSCOhost

| **Search terms** | **Search options** | **Results August 12_2024** | **Search number** |
| --- | --- | --- | --- |
| S4 AND S8 AND S12 | Limiters - Publication Date: 20110101-20241231 | Interface - EBSCOhost Research Databases | 281 |
|  | Expanders - Apply equivalent subjects | Search Screen - Advanced Search |  |
|  | Search modes - Find all my search terms | Database - Academic Search Complete;Atla Historical Monographs Collection: Series 1;Atla Historical Monographs Collection: Series 2;Art & Architecture Source;Atla Religion Database with AtlaSerials PLUS;Australia/New Zealand Reference Centre Plus;Business Book Summaries;Business Source Complete;CINAHL Complete;Communication & Mass Media Complete;Computers & Applied Sciences Complete;eBook Collection (EBSCOhost);EconLit with Full Text;Education Source;Environment Complete;ERIC;GreenFILE;Health Business Elite;Health Source - Consumer Edition;Health Source: Nursing/Academic Edition;Historical Abstracts;Hospitality & Tourism Complete;Humanities International Complete;Legal Source;LGBTQ+ Source;Library, Information Science & Technology Abstracts with Full Text;MAS Ultra - School Edition;MasterFILE Complete;MEDLINE Complete;Mental Measurements Yearbook with Tests in Print;MLA Directory of Periodicals;MLA International Bibliography;New Testament Abstracts;Newspaper Source Plus;Newswires;Philosopher's Index with Full Text;Political Science Complete;APA PsycArticles;APA PsycBooks;APA PsycExtra;Psychology and Behavioral Sciences Collection;APA PsycInfo;APA PsycTests;Regional Business News;Religion and Philosophy Collection;SocINDEX with Full Text;SPORTDiscus with Full Text;Teacher Reference Center;The Serials Directory;MasterFILE Premier Reference eBook Subscription (EBSCOhost);MAS Reference eBook Collection;Literary Reference Source eBook Subscription;OpenDissertations;Literary Reference Plus;Criminal Justice Abstracts with Full Text;Pathways to Research in Business & Economics;Pathways to Research in Education;Australia / New Zealand Reference Centre Plus eBook Subscription (EBSCOhost) |  |
| S4 AND S8 AND S12 | Limiters - Publication Date: 20090101-20241231 | Interface - EBSCOhost Research Databases | 311 |
|  | Expanders - Apply equivalent subjects | Search Screen - Advanced Search |  |
|  | Search modes - Find all my search terms | Database - Academic Search Complete;Atla Historical Monographs Collection: Series 1;Atla Historical Monographs Collection: Series 2;Art & Architecture Source;Atla Religion Database with AtlaSerials PLUS;Australia/New Zealand Reference Centre Plus;Business Book Summaries;Business Source Complete;CINAHL Complete;Communication & Mass Media Complete;Computers & Applied Sciences Complete;eBook Collection (EBSCOhost);EconLit with Full Text;Education Source;Environment Complete;ERIC;GreenFILE;Health Business Elite;Health Source - Consumer Edition;Health Source: Nursing/Academic Edition;Historical Abstracts;Hospitality & Tourism Complete;Humanities International Complete;Legal Source;LGBTQ+ Source;Library, Information Science & Technology Abstracts with Full Text;MAS Ultra - School Edition;MasterFILE Complete;MEDLINE Complete;Mental Measurements Yearbook with Tests in Print;MLA Directory of Periodicals;MLA International Bibliography;New Testament Abstracts;Newspaper Source Plus;Newswires;Philosopher's Index with Full Text;Political Science Complete;APA PsycArticles;APA PsycBooks;APA PsycExtra;Psychology and Behavioral Sciences Collection;APA PsycInfo;APA PsycTests;Regional Business News;Religion and Philosophy Collection;SocINDEX with Full Text;SPORTDiscus with Full Text;Teacher Reference Center;The Serials Directory;MasterFILE Premier Reference eBook Subscription (EBSCOhost);MAS Reference eBook Collection;Literary Reference Source eBook Subscription;OpenDissertations;Literary Reference Plus;Criminal Justice Abstracts with Full Text;Pathways to Research in Business & Economics;Pathways to Research in Education;Australia / New Zealand Reference Centre Plus eBook Subscription (EBSCOhost) |  |
| S4 AND S8 AND S12 | Expanders - Apply equivalent subjects | Interface - EBSCOhost Research Databases | 386 |
|  | Search modes - Find all my search terms | Search Screen - Advanced Search |  |
|  |  | Database - Academic Search Complete;Atla Historical Monographs Collection: Series 1;Atla Historical Monographs Collection: Series 2;Art & Architecture Source;Atla Religion Database with AtlaSerials PLUS;Australia/New Zealand Reference Centre Plus;Business Book Summaries;Business Source Complete;CINAHL Complete;Communication & Mass Media Complete;Computers & Applied Sciences Complete;eBook Collection (EBSCOhost);EconLit with Full Text;Education Source;Environment Complete;ERIC;GreenFILE;Health Business Elite;Health Source - Consumer Edition;Health Source: Nursing/Academic Edition;Historical Abstracts;Hospitality & Tourism Complete;Humanities International Complete;Legal Source;LGBTQ+ Source;Library, Information Science & Technology Abstracts with Full Text;MAS Ultra - School Edition;MasterFILE Complete;MEDLINE Complete;Mental Measurements Yearbook with Tests in Print;MLA Directory of Periodicals;MLA International Bibliography;New Testament Abstracts;Newspaper Source Plus;Newswires;Philosopher's Index with Full Text;Political Science Complete;APA PsycArticles;APA PsycBooks;APA PsycExtra;Psychology and Behavioral Sciences Collection;APA PsycInfo;APA PsycTests;Regional Business News;Religion and Philosophy Collection;SocINDEX with Full Text;SPORTDiscus with Full Text;Teacher Reference Center;The Serials Directory;MasterFILE Premier Reference eBook Subscription (EBSCOhost);MAS Reference eBook Collection;Literary Reference Source eBook Subscription;OpenDissertations;Literary Reference Plus;Criminal Justice Abstracts with Full Text;Pathways to Research in Business & Economics;Pathways to Research in Education;Australia / New Zealand Reference Centre Plus eBook Subscription (EBSCOhost) |  |
| S9 OR S10 OR S11 | Expanders - Apply equivalent subjects | Interface - EBSCOhost Research Databases | 2,120,138 |
|  | Search modes - Find all my search terms | Search Screen - Advanced Search |  |
|  |  | Database - Academic Search Complete;Atla Historical Monographs Collection: Series 1;Atla Historical Monographs Collection: Series 2;Art & Architecture Source;Atla Religion Database with AtlaSerials PLUS;Australia/New Zealand Reference Centre Plus;Business Book Summaries;Business Source Complete;CINAHL Complete;Communication & Mass Media Complete;Computers & Applied Sciences Complete;eBook Collection (EBSCOhost);EconLit with Full Text;Education Source;Environment Complete;ERIC;GreenFILE;Health Business Elite;Health Source - Consumer Edition;Health Source: Nursing/Academic Edition;Historical Abstracts;Hospitality & Tourism Complete;Humanities International Complete;Legal Source;LGBTQ+ Source;Library, Information Science & Technology Abstracts with Full Text;MAS Ultra - School Edition;MasterFILE Complete;MEDLINE Complete;Mental Measurements Yearbook with Tests in Print;MLA Directory of Periodicals;MLA International Bibliography;New Testament Abstracts;Newspaper Source Plus;Newswires;Philosopher's Index with Full Text;Political Science Complete;APA PsycArticles;APA PsycBooks;APA PsycExtra;Psychology and Behavioral Sciences Collection;APA PsycInfo;APA PsycTests;Regional Business News;Religion and Philosophy Collection;SocINDEX with Full Text;SPORTDiscus with Full Text;Teacher Reference Center;The Serials Directory;MasterFILE Premier Reference eBook Subscription (EBSCOhost);MAS Reference eBook Collection;Literary Reference Source eBook Subscription;OpenDissertations;Literary Reference Plus;Criminal Justice Abstracts with Full Text;Pathways to Research in Business & Economics;Pathways to Research in Education;Australia / New Zealand Reference Centre Plus eBook Subscription (EBSCOhost) |  |
| psychological N3 (wellbeing or well being or well-being or wellness or stress* or resilience or state or distress) | Expanders - Apply equivalent subjects | Interface - EBSCOhost Research Databases | 923,023 |
|  | Search modes - Find all my search terms | Search Screen - Advanced Search |  |
|  |  | Database - Academic Search Complete;Atla Historical Monographs Collection: Series 1;Atla Historical Monographs Collection: Series 2;Art & Architecture Source;Atla Religion Database with AtlaSerials PLUS;Australia/New Zealand Reference Centre Plus;Business Book Summaries;Business Source Complete;CINAHL Complete;Communication & Mass Media Complete;Computers & Applied Sciences Complete;eBook Collection (EBSCOhost);EconLit with Full Text;Education Source;Environment Complete;ERIC;GreenFILE;Health Business Elite;Health Source - Consumer Edition;Health Source: Nursing/Academic Edition;Historical Abstracts;Hospitality & Tourism Complete;Humanities International Complete;Legal Source;LGBTQ+ Source;Library, Information Science & Technology Abstracts with Full Text;MAS Ultra - School Edition;MasterFILE Complete;MEDLINE Complete;Mental Measurements Yearbook with Tests in Print;MLA Directory of Periodicals;MLA International Bibliography;New Testament Abstracts;Newspaper Source Plus;Newswires;Philosopher's Index with Full Text;Political Science Complete;APA PsycArticles;APA PsycBooks;APA PsycExtra;Psychology and Behavioral Sciences Collection;APA PsycInfo;APA PsycTests;Regional Business News;Religion and Philosophy Collection;SocINDEX with Full Text;SPORTDiscus with Full Text;Teacher Reference Center;The Serials Directory;MasterFILE Premier Reference eBook Subscription (EBSCOhost);MAS Reference eBook Collection;Literary Reference Source eBook Subscription;OpenDissertations;Literary Reference Plus;Criminal Justice Abstracts with Full Text;Pathways to Research in Business & Economics;Pathways to Research in Education;Australia / New Zealand Reference Centre Plus eBook Subscription (EBSCOhost) |  |
| psychological wellbeing OR psychological state OR psychological distress OR psychological resilience OR psychological stress | Expanders - Apply equivalent subjects | Interface - EBSCOhost Research Databases | 2,019,173 |
|  | Search modes - Find all my search terms | Search Screen - Advanced Search |  |
|  |  | Database - Academic Search Complete;Atla Historical Monographs Collection: Series 1;Atla Historical Monographs Collection: Series 2;Art & Architecture Source;Atla Religion Database with AtlaSerials PLUS;Australia/New Zealand Reference Centre Plus;Business Book Summaries;Business Source Complete;CINAHL Complete;Communication & Mass Media Complete;Computers & Applied Sciences Complete;eBook Collection (EBSCOhost);EconLit with Full Text;Education Source;Environment Complete;ERIC;GreenFILE;Health Business Elite;Health Source - Consumer Edition;Health Source: Nursing/Academic Edition;Historical Abstracts;Hospitality & Tourism Complete;Humanities International Complete;Legal Source;LGBTQ+ Source;Library, Information Science & Technology Abstracts with Full Text;MAS Ultra - School Edition;MasterFILE Complete;MEDLINE Complete;Mental Measurements Yearbook with Tests in Print;MLA Directory of Periodicals;MLA International Bibliography;New Testament Abstracts;Newspaper Source Plus;Newswires;Philosopher's Index with Full Text;Political Science Complete;APA PsycArticles;APA PsycBooks;APA PsycExtra;Psychology and Behavioral Sciences Collection;APA PsycInfo;APA PsycTests;Regional Business News;Religion and Philosophy Collection;SocINDEX with Full Text;SPORTDiscus with Full Text;Teacher Reference Center;The Serials Directory;MasterFILE Premier Reference eBook Subscription (EBSCOhost);MAS Reference eBook Collection;Literary Reference Source eBook Subscription;OpenDissertations;Literary Reference Plus;Criminal Justice Abstracts with Full Text;Pathways to Research in Business & Economics;Pathways to Research in Education;Australia / New Zealand Reference Centre Plus eBook Subscription (EBSCOhost) |  |
| psychological well-being | Expanders - Apply equivalent subjects | Interface - EBSCOhost Research Databases | 426,166 |
|  | Search modes - Find all my search terms | Search Screen - Advanced Search |  |
|  |  | Database - Academic Search Complete;Atla Historical Monographs Collection: Series 1;Atla Historical Monographs Collection: Series 2;Art & Architecture Source;Atla Religion Database with AtlaSerials PLUS;Australia/New Zealand Reference Centre Plus;Business Book Summaries;Business Source Complete;CINAHL Complete;Communication & Mass Media Complete;Computers & Applied Sciences Complete;eBook Collection (EBSCOhost);EconLit with Full Text;Education Source;Environment Complete;ERIC;GreenFILE;Health Business Elite;Health Source - Consumer Edition;Health Source: Nursing/Academic Edition;Historical Abstracts;Hospitality & Tourism Complete;Humanities International Complete;Legal Source;LGBTQ+ Source;Library, Information Science & Technology Abstracts with Full Text;MAS Ultra - School Edition;MasterFILE Complete;MEDLINE Complete;Mental Measurements Yearbook with Tests in Print;MLA Directory of Periodicals;MLA International Bibliography;New Testament Abstracts;Newspaper Source Plus;Newswires;Philosopher's Index with Full Text;Political Science Complete;APA PsycArticles;APA PsycBooks;APA PsycExtra;Psychology and Behavioral Sciences Collection;APA PsycInfo;APA PsycTests;Regional Business News;Religion and Philosophy Collection;SocINDEX with Full Text;SPORTDiscus with Full Text;Teacher Reference Center;The Serials Directory;MasterFILE Premier Reference eBook Subscription (EBSCOhost);MAS Reference eBook Collection;Literary Reference Source eBook Subscription;OpenDissertations;Literary Reference Plus;Criminal Justice Abstracts with Full Text;Pathways to Research in Business & Economics;Pathways to Research in Education;Australia / New Zealand Reference Centre Plus eBook Subscription (EBSCOhost) |  |
| S5 OR S6 OR S7 | Expanders - Apply equivalent subjects | Interface - EBSCOhost Research Databases | 280,844 |
|  | Search modes - Find all my search terms | Search Screen - Advanced Search |  |
|  |  | Database - Academic Search Complete;Atla Historical Monographs Collection: Series 1;Atla Historical Monographs Collection: Series 2;Art & Architecture Source;Atla Religion Database with AtlaSerials PLUS;Australia/New Zealand Reference Centre Plus;Business Book Summaries;Business Source Complete;CINAHL Complete;Communication & Mass Media Complete;Computers & Applied Sciences Complete;eBook Collection (EBSCOhost);EconLit with Full Text;Education Source;Environment Complete;ERIC;GreenFILE;Health Business Elite;Health Source - Consumer Edition;Health Source: Nursing/Academic Edition;Historical Abstracts;Hospitality & Tourism Complete;Humanities International Complete;Legal Source;LGBTQ+ Source;Library, Information Science & Technology Abstracts with Full Text;MAS Ultra - School Edition;MasterFILE Complete;MEDLINE Complete;Mental Measurements Yearbook with Tests in Print;MLA Directory of Periodicals;MLA International Bibliography;New Testament Abstracts;Newspaper Source Plus;Newswires;Philosopher's Index with Full Text;Political Science Complete;APA PsycArticles;APA PsycBooks;APA PsycExtra;Psychology and Behavioral Sciences Collection;APA PsycInfo;APA PsycTests;Regional Business News;Religion and Philosophy Collection;SocINDEX with Full Text;SPORTDiscus with Full Text;Teacher Reference Center;The Serials Directory;MasterFILE Premier Reference eBook Subscription (EBSCOhost);MAS Reference eBook Collection;Literary Reference Source eBook Subscription;OpenDissertations;Literary Reference Plus;Criminal Justice Abstracts with Full Text;Pathways to Research in Business & Economics;Pathways to Research in Education;Australia / New Zealand Reference Centre Plus eBook Subscription (EBSCOhost) |  |
| allergic disease* | Expanders - Apply equivalent subjects | Interface - EBSCOhost Research Databases | 210,148 |
|  | Search modes - Find all my search terms | Search Screen - Advanced Search |  |
|  |  | Database - Academic Search Complete;Atla Historical Monographs Collection: Series 1;Atla Historical Monographs Collection: Series 2;Art & Architecture Source;Atla Religion Database with AtlaSerials PLUS;Australia/New Zealand Reference Centre Plus;Business Book Summaries;Business Source Complete;CINAHL Complete;Communication & Mass Media Complete;Computers & Applied Sciences Complete;eBook Collection (EBSCOhost);EconLit with Full Text;Education Source;Environment Complete;ERIC;GreenFILE;Health Business Elite;Health Source - Consumer Edition;Health Source: Nursing/Academic Edition;Historical Abstracts;Hospitality & Tourism Complete;Humanities International Complete;Legal Source;LGBTQ+ Source;Library, Information Science & Technology Abstracts with Full Text;MAS Ultra - School Edition;MasterFILE Complete;MEDLINE Complete;Mental Measurements Yearbook with Tests in Print;MLA Directory of Periodicals;MLA International Bibliography;New Testament Abstracts;Newspaper Source Plus;Newswires;Philosopher's Index with Full Text;Political Science Complete;APA PsycArticles;APA PsycBooks;APA PsycExtra;Psychology and Behavioral Sciences Collection;APA PsycInfo;APA PsycTests;Regional Business News;Religion and Philosophy Collection;SocINDEX with Full Text;SPORTDiscus with Full Text;Teacher Reference Center;The Serials Directory;MasterFILE Premier Reference eBook Subscription (EBSCOhost);MAS Reference eBook Collection;Literary Reference Source eBook Subscription;OpenDissertations;Literary Reference Plus;Criminal Justice Abstracts with Full Text;Pathways to Research in Business & Economics;Pathways to Research in Education;Australia / New Zealand Reference Centre Plus eBook Subscription (EBSCOhost) |  |
| anaphylaxis OR allergic shock OR severe allergic reaction* OR life threatening allerg* OR allergic emergenc* OR anaphylactic reaction* | Expanders - Apply equivalent subjects | Interface - EBSCOhost Research Databases | 92,453 |
|  | Search modes - Find all my search terms | Search Screen - Advanced Search |  |
|  |  | Database - Academic Search Complete;Atla Historical Monographs Collection: Series 1;Atla Historical Monographs Collection: Series 2;Art & Architecture Source;Atla Religion Database with AtlaSerials PLUS;Australia/New Zealand Reference Centre Plus;Business Book Summaries;Business Source Complete;CINAHL Complete;Communication & Mass Media Complete;Computers & Applied Sciences Complete;eBook Collection (EBSCOhost);EconLit with Full Text;Education Source;Environment Complete;ERIC;GreenFILE;Health Business Elite;Health Source - Consumer Edition;Health Source: Nursing/Academic Edition;Historical Abstracts;Hospitality & Tourism Complete;Humanities International Complete;Legal Source;LGBTQ+ Source;Library, Information Science & Technology Abstracts with Full Text;MAS Ultra - School Edition;MasterFILE Complete;MEDLINE Complete;Mental Measurements Yearbook with Tests in Print;MLA Directory of Periodicals;MLA International Bibliography;New Testament Abstracts;Newspaper Source Plus;Newswires;Philosopher's Index with Full Text;Political Science Complete;APA PsycArticles;APA PsycBooks;APA PsycExtra;Psychology and Behavioral Sciences Collection;APA PsycInfo;APA PsycTests;Regional Business News;Religion and Philosophy Collection;SocINDEX with Full Text;SPORTDiscus with Full Text;Teacher Reference Center;The Serials Directory;MasterFILE Premier Reference eBook Subscription (EBSCOhost);MAS Reference eBook Collection;Literary Reference Source eBook Subscription;OpenDissertations;Literary Reference Plus;Criminal Justice Abstracts with Full Text;Pathways to Research in Business & Economics;Pathways to Research in Education;Australia / New Zealand Reference Centre Plus eBook Subscription (EBSCOhost) |  |
| anaphylaxis | Expanders - Apply equivalent subjects | Interface - EBSCOhost Research Databases | 64,281 |
|  | Search modes - Find all my search terms | Search Screen - Advanced Search |  |
|  |  | Database - Academic Search Complete;Atla Historical Monographs Collection: Series 1;Atla Historical Monographs Collection: Series 2;Art & Architecture Source;Atla Religion Database with AtlaSerials PLUS;Australia/New Zealand Reference Centre Plus;Business Book Summaries;Business Source Complete;CINAHL Complete;Communication & Mass Media Complete;Computers & Applied Sciences Complete;eBook Collection (EBSCOhost);EconLit with Full Text;Education Source;Environment Complete;ERIC;GreenFILE;Health Business Elite;Health Source - Consumer Edition;Health Source: Nursing/Academic Edition;Historical Abstracts;Hospitality & Tourism Complete;Humanities International Complete;Legal Source;LGBTQ+ Source;Library, Information Science & Technology Abstracts with Full Text;MAS Ultra - School Edition;MasterFILE Complete;MEDLINE Complete;Mental Measurements Yearbook with Tests in Print;MLA Directory of Periodicals;MLA International Bibliography;New Testament Abstracts;Newspaper Source Plus;Newswires;Philosopher's Index with Full Text;Political Science Complete;APA PsycArticles;APA PsycBooks;APA PsycExtra;Psychology and Behavioral Sciences Collection;APA PsycInfo;APA PsycTests;Regional Business News;Religion and Philosophy Collection;SocINDEX with Full Text;SPORTDiscus with Full Text;Teacher Reference Center;The Serials Directory;MasterFILE Premier Reference eBook Subscription (EBSCOhost);MAS Reference eBook Collection;Literary Reference Source eBook Subscription;OpenDissertations;Literary Reference Plus;Criminal Justice Abstracts with Full Text;Pathways to Research in Business & Economics;Pathways to Research in Education;Australia / New Zealand Reference Centre Plus eBook Subscription (EBSCOhost) |  |
| S1 OR S2 OR S3 | Expanders - Apply equivalent subjects | Interface - EBSCOhost Research Databases | 2,946,209 |
|  | Search modes - Find all my search terms | Search Screen - Advanced Search |  |
|  |  | Database - Academic Search Complete;Atla Historical Monographs Collection: Series 1;Atla Historical Monographs Collection: Series 2;Art & Architecture Source;Atla Religion Database with AtlaSerials PLUS;Australia/New Zealand Reference Centre Plus;Business Book Summaries;Business Source Complete;CINAHL Complete;Communication & Mass Media Complete;Computers & Applied Sciences Complete;eBook Collection (EBSCOhost);EconLit with Full Text;Education Source;Environment Complete;ERIC;GreenFILE;Health Business Elite;Health Source - Consumer Edition;Health Source: Nursing/Academic Edition;Historical Abstracts;Hospitality & Tourism Complete;Humanities International Complete;Legal Source;LGBTQ+ Source;Library, Information Science & Technology Abstracts with Full Text;MAS Ultra - School Edition;MasterFILE Complete;MEDLINE Complete;Mental Measurements Yearbook with Tests in Print;MLA Directory of Periodicals;MLA International Bibliography;New Testament Abstracts;Newspaper Source Plus;Newswires;Philosopher's Index with Full Text;Political Science Complete;APA PsycArticles;APA PsycBooks;APA PsycExtra;Psychology and Behavioral Sciences Collection;APA PsycInfo;APA PsycTests;Regional Business News;Religion and Philosophy Collection;SocINDEX with Full Text;SPORTDiscus with Full Text;Teacher Reference Center;The Serials Directory;MasterFILE Premier Reference eBook Subscription (EBSCOhost);MAS Reference eBook Collection;Literary Reference Source eBook Subscription;OpenDissertations;Literary Reference Plus;Criminal Justice Abstracts with Full Text;Pathways to Research in Business & Economics;Pathways to Research in Education;Australia / New Zealand Reference Centre Plus eBook Subscription (EBSCOhost) |  |
| quality of life OR wellbeing OR well-being OR health related quality of life OR QOL* OR HRQOL* | Expanders - Apply equivalent subjects | Interface - EBSCOhost Research Databases | 2,946,312 |
|  | Search modes - Proximity | Search Screen - Advanced Search |  |
|  |  | Database - Academic Search Complete;Atla Historical Monographs Collection: Series 1;Atla Historical Monographs Collection: Series 2;Art & Architecture Source;Atla Religion Database with AtlaSerials PLUS;Australia/New Zealand Reference Centre Plus;Business Book Summaries;Business Source Complete;CINAHL Complete;Communication & Mass Media Complete;Computers & Applied Sciences Complete;eBook Collection (EBSCOhost);EconLit with Full Text;Education Source;Environment Complete;ERIC;GreenFILE;Health Business Elite;Health Source - Consumer Edition;Health Source: Nursing/Academic Edition;Historical Abstracts;Hospitality & Tourism Complete;Humanities International Complete;Legal Source;LGBTQ+ Source;Library, Information Science & Technology Abstracts with Full Text;MAS Ultra - School Edition;MasterFILE Complete;MEDLINE Complete;Mental Measurements Yearbook with Tests in Print;MLA Directory of Periodicals;MLA International Bibliography;New Testament Abstracts;Newspaper Source Plus;Newswires;Philosopher's Index with Full Text;Political Science Complete;APA PsycArticles;APA PsycBooks;APA PsycExtra;Psychology and Behavioral Sciences Collection;APA PsycInfo;APA PsycTests;Regional Business News;Religion and Philosophy Collection;SocINDEX with Full Text;SPORTDiscus with Full Text;Teacher Reference Center;The Serials Directory;MasterFILE Premier Reference eBook Subscription (EBSCOhost);MAS Reference eBook Collection;Literary Reference Source eBook Subscription;OpenDissertations;Literary Reference Plus;Criminal Justice Abstracts with Full Text;Pathways to Research in Business & Economics;Pathways to Research in Education;Australia / New Zealand Reference Centre Plus eBook Subscription (EBSCOhost) |  |
| quality N3 life | Expanders - Apply equivalent subjects | Interface - EBSCOhost Research Databases | 1,656,000 |
|  | Search modes - Proximity | Search Screen - Advanced Search |  |
|  |  | Database - Academic Search Complete;Atla Historical Monographs Collection: Series 1;Atla Historical Monographs Collection: Series 2;Art & Architecture Source;Atla Religion Database with AtlaSerials PLUS;Australia/New Zealand Reference Centre Plus;Business Book Summaries;Business Source Complete;CINAHL Complete;Communication & Mass Media Complete;Computers & Applied Sciences Complete;eBook Collection (EBSCOhost);EconLit with Full Text;Education Source;Environment Complete;ERIC;GreenFILE;Health Business Elite;Health Source - Consumer Edition;Health Source: Nursing/Academic Edition;Historical Abstracts;Hospitality & Tourism Complete;Humanities International Complete;Legal Source;LGBTQ+ Source;Library, Information Science & Technology Abstracts with Full Text;MAS Ultra - School Edition;MasterFILE Complete;MEDLINE Complete;Mental Measurements Yearbook with Tests in Print;MLA Directory of Periodicals;MLA International Bibliography;New Testament Abstracts;Newspaper Source Plus;Newswires;Philosopher's Index with Full Text;Political Science Complete;APA PsycArticles;APA PsycBooks;APA PsycExtra;Psychology and Behavioral Sciences Collection;APA PsycInfo;APA PsycTests;Regional Business News;Religion and Philosophy Collection;SocINDEX with Full Text;SPORTDiscus with Full Text;Teacher Reference Center;The Serials Directory;MasterFILE Premier Reference eBook Subscription (EBSCOhost);MAS Reference eBook Collection;Literary Reference Source eBook Subscription;OpenDissertations;Literary Reference Plus;Criminal Justice Abstracts with Full Text;Pathways to Research in Business & Economics;Pathways to Research in Education;Australia / New Zealand Reference Centre Plus eBook Subscription (EBSCOhost) |  |
| quality of life OR psychological well-being | Expanders - Apply equivalent subjects | Interface - EBSCOhost Research Databases | 1,987,266 |
|  | Search modes - Proximity | Search Screen - Advanced Search |  |
|  |  | Database - Academic Search Complete;Atla Historical Monographs Collection: Series 1;Atla Historical Monographs Collection: Series 2;Art & Architecture Source;Atla Religion Database with AtlaSerials PLUS;Australia/New Zealand Reference Centre Plus;Business Book Summaries;Business Source Complete;CINAHL Complete;Communication & Mass Media Complete;Computers & Applied Sciences Complete;eBook Collection (EBSCOhost);EconLit with Full Text;Education Source;Environment Complete;ERIC;GreenFILE;Health Business Elite;Health Source - Consumer Edition;Health Source: Nursing/Academic Edition;Historical Abstracts;Hospitality & Tourism Complete;Humanities International Complete;Legal Source;LGBTQ+ Source;Library, Information Science & Technology Abstracts with Full Text;MAS Ultra - School Edition;MasterFILE Complete;MEDLINE Complete;Mental Measurements Yearbook with Tests in Print;MLA Directory of Periodicals;MLA International Bibliography;New Testament Abstracts;Newspaper Source Plus;Newswires;Philosopher's Index with Full Text;Political Science Complete;APA PsycArticles;APA PsycBooks;APA PsycExtra;Psychology and Behavioral Sciences Collection;APA PsycInfo;APA PsycTests;Regional Business News;Religion and Philosophy Collection;SocINDEX with Full Text;SPORTDiscus with Full Text;Teacher Reference Center;The Serials Directory;MasterFILE Premier Reference eBook Subscription (EBSCOhost);MAS Reference eBook Collection;Literary Reference Source eBook Subscription;OpenDissertations;Literary Reference Plus;Criminal Justice Abstracts with Full Text;Pathways to Research in Business & Economics;Pathways to Research in Education;Australia / New Zealand Reference Centre Plus eBook Subscription (EBSCOhost) |  |

MEDLINE

| **Search number** | **Search terms** | **Search options** | **Results August 22_2024** | **Search number** |
| --- | --- | --- | --- | --- |
| S14 | S4 AND S8 AND S12 | Limiters - Publication Date: 20110101-20231231 | Interface - EBSCOhost Research Databases | 36 |
|  |  | Expanders - Apply equivalent subjects | Search Screen - Advanced Search |  |
|  |  | Search modes - Find all my search terms | Database - MEDLINE Complete |  |
| S13 | S4 AND S8 AND S12 | Expanders - Apply equivalent subjects | Interface - EBSCOhost Research Databases | 44 |
|  |  | Search modes - Find all my search terms | Search Screen - Advanced Search |  |
|  |  |  | Database - MEDLINE Complete |  |
| S12 | S9 OR S10 OR S11 | Expanders - Apply equivalent subjects | Interface - EBSCOhost Research Databases | 187,225 |
|  |  | Search modes - Find all my search terms | Search Screen - Advanced Search |  |
|  |  |  | Database - MEDLINE Complete |  |
| S11 | "psychological N3 (wellbeing or well being or well-being or wellness or stress* or resilience or state or distress) " | Expanders - Apply equivalent subjects | Interface - EBSCOhost Research Databases | 16,557 |
|  |  | Search modes - SmartText Searching | Search Screen - Advanced Search |  |
|  |  |  | Database - MEDLINE Complete |  |
| S10 | "psychological well-being" OR "psychological state" OR "Psychological distress" OR "Psychological resilience" OR "Psychological stress" | Expanders - Apply equivalent subjects | Interface - EBSCOhost Research Databases | 183,627 |
|  |  | Search modes - Proximity | Search Screen - Advanced Search |  |
|  |  |  | Database - MEDLINE Complete |  |
| S9 | "psychological wellbeing" | Expanders - Apply equivalent subjects | Interface - EBSCOhost Research Databases | 3,163 |
|  |  | Search modes - Proximity | Search Screen - Advanced Search |  |
|  |  |  | Database - MEDLINE Complete |  |
| S8 | S5 OR S6 OR S7 | Expanders - Apply equivalent subjects | Interface - EBSCOhost Research Databases | 90,855 |
|  |  | Search modes - Find all my search terms | Search Screen - Advanced Search |  |
|  |  |  | Database - MEDLINE Complete |  |
| S7 | "allergic disease*" | Expanders - Apply equivalent subjects | Interface - EBSCOhost Research Databases | 58,781 |
|  |  | Search modes - Proximity | Search Screen - Advanced Search |  |
|  |  |  | Database - MEDLINE Complete |  |
| S6 | "Anaphylaxis" OR "Allergic shock" OR "Severe allergic reaction*" OR "life threatening allerg*" OR "Allergic emergenc*" OR "Anaphylactic reaction*" | Expanders - Apply equivalent subjects | Interface - EBSCOhost Research Databases | 37,616 |
|  |  | Search modes - Proximity | Search Screen - Advanced Search |  |
|  |  |  | Database - MEDLINE Complete |  |
| S5 | "Anaphylaxis" | Expanders - Apply equivalent subjects | Interface - EBSCOhost Research Databases | 35,130 |
|  |  | Search modes - Proximity | Search Screen - Advanced Search |  |
|  |  |  | Database - MEDLINE Complete |  |
| S4 | S1 OR S2 OR S3 | Expanders - Apply equivalent subjects | Interface - EBSCOhost Research Databases | 668,203 |
|  |  | Search modes - Find all my search terms | Search Screen - Advanced Search |  |
|  |  |  | Database - MEDLINE Complete |  |
| S3 | "Quality of life" OR "wellbeing" OR "well-being" OR "health related quality of life" OR "QOL*" OR "HRQOL*" | Expanders - Apply equivalent subjects | Interface - EBSCOhost Research Databases | 650,217 |
|  |  | Search modes - Proximity | Search Screen - Advanced Search |  |
|  |  |  | Database - MEDLINE Complete |  |
| S2 | "Quality N3 life" | Expanders - Apply equivalent subjects | Interface - EBSCOhost Research Databases | 808,552 |
|  |  | Search modes - SmartText Searching | Search Screen - Advanced Search |  |
|  |  |  | Database - MEDLINE Complete |  |
| S1 | "Quality of life" OR "Psychological well-being" | Expanders - Apply equivalent subjects | Interface - EBSCOhost Research Databases | 511,582 |
|  |  | Search modes - Proximity | Search Screen - Advanced Search |  |
|  |  |  | Database - MEDLINE Complete |  |

CINAHL Complete

| **Search number** | **Search terms** | **Search options** | **Results August 22_2024** | **Search number** |
| --- | --- | --- | --- | --- |
| S15 | S4 AND S8 AND S13 | Limiters - Publication Date: 20110101-20241231 | Interface - EBSCOhost Research Databases | 15 |
|  |  | Expanders - Apply equivalent subjects | Search Screen - Advanced Search |  |
|  |  | Search modes - Find all my search terms | Database - CINAHL Complete |  |
| S14 | S4 AND S8 AND S13 | Expanders - Apply equivalent subjects | Interface - EBSCOhost Research Databases | 16 |
|  |  | Search modes - Find all my search terms | Search Screen - Advanced Search |  |
|  |  |  | Database - CINAHL Complete |  |
| S13 | S9 OR S10 OR S11 OR S12 | Expanders - Apply equivalent subjects | Interface - EBSCOhost Research Databases | 115,920 |
|  |  | Search modes - Find all my search terms | Search Screen - Advanced Search |  |
|  |  |  | Database - CINAHL Complete |  |
| S12 | "psychological N3 (wellbeing or well being or well-being or wellness or stress* or resilience or state or distress) " | Expanders - Apply equivalent subjects | Interface - EBSCOhost Research Databases | 13,742 |
|  |  | Search modes - SmartText Searching | Search Screen - Advanced Search |  |
|  |  |  | Database - CINAHL Complete |  |
| S11 | "psychological well-being" OR "Psychological stress" OR "psychological state" OR "Psychological distress" OR "Psychological resilience" | Expanders - Apply equivalent subjects | Interface - EBSCOhost Research Databases | 115,238 |
|  |  | Search modes - Proximity | Search Screen - Advanced Search |  |
|  |  |  | Database - CINAHL Complete |  |
| S10 | "psychological well-being" AND "Psychological stress" AND "psychological state" AND "Psychological distress" AND "Psychological resilience" | Expanders - Apply equivalent subjects | Interface - EBSCOhost Research Databases | 0 |
|  |  | Search modes - Find all my search terms | Search Screen - Advanced Search |  |
|  |  |  | Database - CINAHL Complete |  |
| S9 | "Psychological wellbeing" | Expanders - Apply equivalent subjects | Interface - EBSCOhost Research Databases | 20,702 |
|  |  | Search modes - Find all my search terms | Search Screen - Advanced Search |  |
|  |  |  | Database - CINAHL Complete |  |
| S8 | S5 OR S6 OR S7 | Expanders - Apply equivalent subjects | Interface - EBSCOhost Research Databases | 13,657 |
|  |  | Search modes - Find all my search terms | Search Screen - Advanced Search |  |
|  |  |  | Database - CINAHL Complete |  |
| S7 | "allergic disease*" | Expanders - Apply equivalent subjects | Interface - EBSCOhost Research Databases | 7,711 |
|  |  | Search modes - Find all my search terms | Search Screen - Advanced Search |  |
|  |  |  | Database - CINAHL Complete |  |
| S6 | "Anaphylaxis" OR "Anaphylactic reaction*" OR "Allergic shock" OR "Severe allergic reaction*" OR "life threatening allerg*" OR "Allergic emergenc*" | Expanders - Apply equivalent subjects | Interface - EBSCOhost Research Databases | 6,474 |
|  |  | Search modes - Find all my search terms | Search Screen - Advanced Search |  |
|  |  |  | Database - CINAHL Complete |  |
| S5 | "Anaphylaxis" | Expanders - Apply equivalent subjects | Interface - EBSCOhost Research Databases | 6,085 |
|  |  | Search modes - Find all my search terms | Search Screen - Advanced Search |  |
|  |  |  | Database - CINAHL Complete |  |
| S4 | S1 OR S2 OR S3 | Expanders - Apply equivalent subjects | Interface - EBSCOhost Research Databases | 338,651 |
|  |  | Search modes - Find all my search terms | Search Screen - Advanced Search |  |
|  |  |  | Database - CINAHL Complete |  |
| S3 | "Quality of life" OR "HRQOL*" OR "wellbeing" OR "well-being" OR "health related quality of life" OR "QOL*" | Expanders - Apply equivalent subjects | Interface - EBSCOhost Research Databases | 333,113 |
|  |  | Search modes - Proximity | Search Screen - Advanced Search |  |
|  |  |  | Database - CINAHL Complete |  |
| S2 | Quality N3 life | Expanders - Apply equivalent subjects | Interface - EBSCOhost Research Databases | 252,276 |
|  |  | Search modes - Proximity | Search Screen - Advanced Search |  |
|  |  |  | Database - CINAHL Complete |  |
| S1 | "Quality of life" OR "Psychological well-being" | Expanders - Apply equivalent subjects | Interface - EBSCOhost Research Databases | 286,555 |
|  |  | Search modes - Proximity | Search Screen - Advanced Search |  |
|  |  |  | Database - CINAHL Complete |  |

Joanna Briggs Institute [JBI]

| **Search number** | **Search terms** | **Search number results 22nd of August** |
| --- | --- | --- |
| 13 | 4 and 8 and 12 | 0 |
|  |  |  |
| 12 | 9 or 10 or 11 | 323 |
|  |  |  |
| 11 | "psychological N3 (wellbeing or well being or well-being or wellness or stress* or resilience or state or distress) ".af. | 0 |
|  |  |  |
| 10 | (("psychological well-being" and "psychological state") or "Psychological distress" or "Psychological resilience" or "Psychological stress").af. | 289 |
|  |  |  |
| 9 | "psychological wellbeing".af. | 62 |
|  |  |  |
| 8 | 5 or 6 or 7 | 52 |
|  |  |  |
| 7 | "allergic disease*".af. | 9 |
|  |  |  |
| 6 | ("Anaphylaxis" or "Allergic shock" or "Severe allergic reaction*" or "life threatening allerg*" or "Anaphylactic reaction*").af. | 46 |
|  |  |  |
| 5 | "Anaphylaxis".af. | 41 |
|  |  |  |
| 4 | 1 or 2 or 3 | 2401 |
|  |  |  |
| 3 | (("Quality of life" and "wellbeing") or "well-being" or "health related quality of life" or "QOL*" or "HRQOL*").af. | 1358 |
|  |  |  |
| 2 | "Quality N3 life".mp. [mp=text, heading word, subject area node word, title] | 0 |
|  |  |  |
| 1 | ("Quality of life" or "Psychological well-being").mp. [mp=text, heading word, subject area node word, title] | 2064 |

Nursing and Allied Health ProQuest

| **S21** | [[S7] AND [S16] AND [S18]Limits applied](https://www.proquest.com/recentsearches.recentsearchtabview.recentsearchesgridview.scrolledrecentsearchlist.checkdbssearchlink:rerunsearch/394EBBEE0494427CPQ/None/$N?site=nahs&t:ac=RecentSearches) | Nursing & Allied Health Database | [2,922](https://www.proquest.com/recentsearches.recentsearchtabview.recentsearchesgridview.scrolledrecentsearchlist.checkdbssearchlink_0:rerunsearch/394EBBEE0494427CPQ/None/$N?site=nahs&t:ac=RecentSearches) |
| --- | --- | --- | --- |
| **S20** | [[S7] AND [S16] AND [S18]Limits applied](https://www.proquest.com/recentsearches.recentsearchtabview.recentsearchesgridview.scrolledrecentsearchlist.checkdbssearchlink:rerunsearch/B7EAE0E17E2B42AAPQ/None/$N?site=nahs&t:ac=RecentSearches) | Nursing & Allied Health Database | [3,912](https://www.proquest.com/recentsearches.recentsearchtabview.recentsearchesgridview.scrolledrecentsearchlist.checkdbssearchlink_0:rerunsearch/B7EAE0E17E2B42AAPQ/None/$N?site=nahs&t:ac=RecentSearches) |
| **S19** | [[S7] AND [S16] AND [S18]](https://www.proquest.com/recentsearches.recentsearchtabview.recentsearchesgridview.scrolledrecentsearchlist.checkdbssearchlink:rerunsearch/B1B6BD0E72974566PQ/None/$N?site=nahs&t:ac=RecentSearches) | Nursing & Allied Health Database | [6,748](https://www.proquest.com/recentsearches.recentsearchtabview.recentsearchesgridview.scrolledrecentsearchlist.checkdbssearchlink_0:rerunsearch/B1B6BD0E72974566PQ/None/$N?site=nahs&t:ac=RecentSearches) |
| **S18** | [[S8] OR [S13] OR [S14] OR [S17]](https://www.proquest.com/recentsearches.recentsearchtabview.recentsearchesgridview.scrolledrecentsearchlist.checkdbssearchlink:rerunsearch/B2BADA98A89C4025PQ/None/$N?site=nahs&t:ac=RecentSearches) | Nursing & Allied Health Database | [149,371](https://www.proquest.com/recentsearches.recentsearchtabview.recentsearchesgridview.scrolledrecentsearchlist.checkdbssearchlink_0:rerunsearch/B2BADA98A89C4025PQ/None/$N?site=nahs&t:ac=RecentSearches) |
| **S17** | ["psychological" NEAR/3 ("wellbeing" or "well being" or "well-being" or "wellness or stress*" or "resilience" or "state" or "distress")](https://www.proquest.com/recentsearches.recentsearchtabview.recentsearchesgridview.scrolledrecentsearchlist.checkdbssearchlink:rerunsearch/877E197952614045PQ/None/$N?site=nahs&t:ac=RecentSearches) | Nursing & Allied Health Database | [112,760](https://www.proquest.com/recentsearches.recentsearchtabview.recentsearchesgridview.scrolledrecentsearchlist.checkdbssearchlink_0:rerunsearch/877E197952614045PQ/None/$N?site=nahs&t:ac=RecentSearches) |
| **S16** | [[S8] OR [S10] OR [S15]](https://www.proquest.com/recentsearches.recentsearchtabview.recentsearchesgridview.scrolledrecentsearchlist.checkdbssearchlink:rerunsearch/41679B72FE6C4B52PQ/None/$N?site=nahs&t:ac=RecentSearches) | Nursing & Allied Health Database | [41,724](https://www.proquest.com/recentsearches.recentsearchtabview.recentsearchesgridview.scrolledrecentsearchlist.checkdbssearchlink_0:rerunsearch/41679B72FE6C4B52PQ/None/$N?site=nahs&t:ac=RecentSearches) |
| **S15** | ["Anaphylaxis" OR "Allergic shock" OR "Severe allergic reaction*" OR ("life-threatening" NEAR/3 allerg*) OR "Allergic emergenc*" OR "Anaphylactic reaction*"](https://www.proquest.com/recentsearches.recentsearchtabview.recentsearchesgridview.scrolledrecentsearchlist.checkdbssearchlink:rerunsearch/9A09614D52DD417DPQ/None/$N?site=nahs&t:ac=RecentSearches) | Nursing & Allied Health Database | [27,506](https://www.proquest.com/recentsearches.recentsearchtabview.recentsearchesgridview.scrolledrecentsearchlist.checkdbssearchlink_0:rerunsearch/9A09614D52DD417DPQ/None/$N?site=nahs&t:ac=RecentSearches) |
| **S14** | ["psychological well-being" OR "psychological state" OR "Psychological distress" OR "Psychological resilience" OR "Psychological stress"](https://www.proquest.com/recentsearches.recentsearchtabview.recentsearchesgridview.scrolledrecentsearchlist.checkdbssearchlink:rerunsearch/1FB6EA90FD254FA0PQ/None/$N?site=nahs&t:ac=RecentSearches) | Nursing & Allied Health Database | [113,636](https://www.proquest.com/recentsearches.recentsearchtabview.recentsearchesgridview.scrolledrecentsearchlist.checkdbssearchlink_0:rerunsearch/1FB6EA90FD254FA0PQ/None/$N?site=nahs&t:ac=RecentSearches) |
| **S13** | ["psychological wellbeing"](https://www.proquest.com/recentsearches.recentsearchtabview.recentsearchesgridview.scrolledrecentsearchlist.checkdbssearchlink:rerunsearch/C5C1DEAE3AA64223PQ/None/$N?site=nahs&t:ac=RecentSearches) | Nursing & Allied Health Database | [9,486](https://www.proquest.com/recentsearches.recentsearchtabview.recentsearchesgridview.scrolledrecentsearchlist.checkdbssearchlink_0:rerunsearch/C5C1DEAE3AA64223PQ/None/$N?site=nahs&t:ac=RecentSearches) |
| **S12** | [[S8] OR [S10] OR [S11]](https://www.proquest.com/recentsearches.recentsearchtabview.recentsearchesgridview.scrolledrecentsearchlist.checkdbssearchlink:rerunsearch/91E7E7FD44B64A5BPQ/None/$N?site=nahs&t:ac=RecentSearches) | Nursing & Allied Health Database | [41,469](https://www.proquest.com/recentsearches.recentsearchtabview.recentsearchesgridview.scrolledrecentsearchlist.checkdbssearchlink_0:rerunsearch/91E7E7FD44B64A5BPQ/None/$N?site=nahs&t:ac=RecentSearches) |
| **S11** | ["Anaphylaxis" OR "Allergic shock" OR "Severe allergic reaction*" OR "life threatening allerg*" OR "Allergic emergenc*" OR "Anaphylactic reaction*"](https://www.proquest.com/recentsearches.recentsearchtabview.recentsearchesgridview.scrolledrecentsearchlist.checkdbssearchlink:rerunsearch/F7BEE486FD504577PQ/None/$N?site=nahs&t:ac=RecentSearches) | Nursing & Allied Health Database | [27,218](https://www.proquest.com/recentsearches.recentsearchtabview.recentsearchesgridview.scrolledrecentsearchlist.checkdbssearchlink_0:rerunsearch/F7BEE486FD504577PQ/None/$N?site=nahs&t:ac=RecentSearches) |
| **S10** | ["allergic disease*"](https://www.proquest.com/recentsearches.recentsearchtabview.recentsearchesgridview.scrolledrecentsearchlist.checkdbssearchlink:rerunsearch/7D1F6B96B3CF4D2DPQ/None/$N?site=nahs&t:ac=RecentSearches) | Nursing & Allied Health Database | [17,521](https://www.proquest.com/recentsearches.recentsearchtabview.recentsearchesgridview.scrolledrecentsearchlist.checkdbssearchlink_0:rerunsearch/7D1F6B96B3CF4D2DPQ/None/$N?site=nahs&t:ac=RecentSearches) |
| **S9** | ["Anaphylaxis" OR "Allergic shock" OR "Severe allergic reaction*" OR "life threatening allerg*" OR "Allergic emergenc*" AND "Anaphylactic reaction*"](https://www.proquest.com/recentsearches.recentsearchtabview.recentsearchesgridview.scrolledrecentsearchlist.checkdbssearchlink:rerunsearch/CE902C1DCA9042FFPQ/None/$N?site=nahs&t:ac=RecentSearches) | Nursing & Allied Health Database | [24,064](https://www.proquest.com/recentsearches.recentsearchtabview.recentsearchesgridview.scrolledrecentsearchlist.checkdbssearchlink_0:rerunsearch/CE902C1DCA9042FFPQ/None/$N?site=nahs&t:ac=RecentSearches) |
| **S8** | ["Anaphylaxis"](https://www.proquest.com/recentsearches.recentsearchtabview.recentsearchesgridview.scrolledrecentsearchlist.checkdbssearchlink:rerunsearch/C721FE0DE30E4DC1PQ/None/$N?site=nahs&t:ac=RecentSearches) | Nursing & Allied Health Database | [21,820](https://www.proquest.com/recentsearches.recentsearchtabview.recentsearchesgridview.scrolledrecentsearchlist.checkdbssearchlink_0:rerunsearch/C721FE0DE30E4DC1PQ/None/$N?site=nahs&t:ac=RecentSearches) |
| **S7** | [[S2] OR [S4] OR [S6]](https://www.proquest.com/recentsearches.recentsearchtabview.recentsearchesgridview.scrolledrecentsearchlist.checkdbssearchlink:rerunsearch/BCCCD18201B8433CPQ/None/$N?site=nahs&t:ac=RecentSearches) | Nursing & Allied Health Database | [1,041,591](https://www.proquest.com/recentsearches.recentsearchtabview.recentsearchesgridview.scrolledrecentsearchlist.checkdbssearchlink_0:rerunsearch/BCCCD18201B8433CPQ/None/$N?site=nahs&t:ac=RecentSearches) |
| **S6** | ["Quality of life" OR "wellbeing" OR "well-being" OR "health related quality of life" OR "QOL*" OR "HRQOL*"](https://www.proquest.com/recentsearches.recentsearchtabview.recentsearchesgridview.scrolledrecentsearchlist.checkdbssearchlink:rerunsearch/B5073AD86EF14D18PQ/None/$N?site=nahs&t:ac=RecentSearches) | Nursing & Allied Health Database | [656,579](https://www.proquest.com/recentsearches.recentsearchtabview.recentsearchesgridview.scrolledrecentsearchlist.checkdbssearchlink_0:rerunsearch/B5073AD86EF14D18PQ/None/$N?site=nahs&t:ac=RecentSearches) |
| **S5** | ["Quality of life" OR "wellbeing" OR "well-being" OR "health related quality of life" AND "QOL*" AND "HRQOL*"](https://www.proquest.com/recentsearches.recentsearchtabview.recentsearchesgridview.scrolledrecentsearchlist.checkdbssearchlink:rerunsearch/2C4EF082C1AD4D52PQ/None/$N?site=nahs&t:ac=RecentSearches) | Nursing & Allied Health Database | [655,332](https://www.proquest.com/recentsearches.recentsearchtabview.recentsearchesgridview.scrolledrecentsearchlist.checkdbssearchlink_0:rerunsearch/2C4EF082C1AD4D52PQ/None/$N?site=nahs&t:ac=RecentSearches) |
| **S4** | [quality NEAR/3 life](https://www.proquest.com/recentsearches.recentsearchtabview.recentsearchesgridview.scrolledrecentsearchlist.checkdbssearchlink:rerunsearch/65295DC2ED6B437CPQ/None/$N?site=nahs&t:ac=RecentSearches) | Nursing & Allied Health Database | [457,615](https://www.proquest.com/recentsearches.recentsearchtabview.recentsearchesgridview.scrolledrecentsearchlist.checkdbssearchlink_0:rerunsearch/65295DC2ED6B437CPQ/None/$N?site=nahs&t:ac=RecentSearches) |
| **S3** | [(quality NEAR/3 life) OR (psychological well-being)](https://www.proquest.com/recentsearches.recentsearchtabview.recentsearchesgridview.scrolledrecentsearchlist.checkdbssearchlink:rerunsearch/39686C873319430CPQ/None/$N?site=nahs&t:ac=RecentSearches) | Nursing & Allied Health Database | [529,794](https://www.proquest.com/recentsearches.recentsearchtabview.recentsearchesgridview.scrolledrecentsearchlist.checkdbssearchlink_0:rerunsearch/39686C873319430CPQ/None/$N?site=nahs&t:ac=RecentSearches) |
| **S2** | [(quality of life) OR (psychological well-being)](https://www.proquest.com/recentsearches.recentsearchtabview.recentsearchesgridview.scrolledrecentsearchlist.checkdbssearchlink:rerunsearch/5C382C86BD83420EPQ/None/$N?site=nahs&t:ac=RecentSearches) | Nursing & Allied Health Database | [935,008](https://www.proquest.com/recentsearches.recentsearchtabview.recentsearchesgridview.scrolledrecentsearchlist.checkdbssearchlink_0:rerunsearch/5C382C86BD83420EPQ/None/$N?site=nahs&t:ac=RecentSearches) |

PsycINFO

| **Search number** | **Search terms** | **Search Options** | **Results** |
| --- | --- | --- | --- |
| S14 | S34 AND S38 AND S42 | **Limiters** - Publication Year: 2011-2024 | 22 |
|  |  | **Expanders** - Apply equivalent subjects |  |
|  |  | **Search modes** - Find all my search terms |  |
| S13 | S34 AND S38 AND S42 | **Expanders** - Apply equivalent subjects | 28 |
|  |  | **Search modes** - Find all my search terms |  |
|  |  |  |  |
| S12 | S39 OR S40 OR S41 | **Expanders** - Apply equivalent subjects | [464,428](javascript:__doPostBack('ctl00$ctl00$FindField$FindField$historyControl$HistoryRepeater$ctl02$linkResults','')) |
|  |  | **Search modes** - Find all my search terms |  |
| S11 | psychological N3 (wellbeing or well being or well-being or wellness or stress* or resilience or state or distress) | **Expanders** - Apply equivalent subjects | [140,163](javascript:__doPostBack('ctl00$ctl00$FindField$FindField$historyControl$HistoryRepeater$ctl03$linkResults','')) |
|  |  | **Search modes** - Proximity |  |
| S10 | psychological wellbeing OR psychological state OR psychological distress OR psychological resilience OR psychological stress | **Expanders** - Apply equivalent subjects | [449,298](javascript:__doPostBack('ctl00$ctl00$FindField$FindField$historyControl$HistoryRepeater$ctl04$linkResults','')) |
|  |  | **Search modes** - Find all my search terms |  |
| S9 | psychological well-being | **Expanders** - Apply equivalent subjects | [85,053](javascript:__doPostBack('ctl00$ctl00$FindField$FindField$historyControl$HistoryRepeater$ctl05$linkResults','')) |
|  |  | **Search modes** - Find all my search terms |  |
| S8 | S35 OR S36 OR S37 | **Expanders** - Apply equivalent subjects | [1,093](javascript:__doPostBack('ctl00$ctl00$FindField$FindField$historyControl$HistoryRepeater$ctl06$linkResults','')) |
|  |  | **Search modes** - Find all my search terms |  |
| S7 | allergic disease* | **Expanders** - Apply equivalent subjects | [834](javascript:__doPostBack('ctl00$ctl00$FindField$FindField$historyControl$HistoryRepeater$ctl07$linkResults','')) |
|  |  | **Search modes** - Proximity |  |
| S6 | anaphylaxis OR allergic shock OR severe allergic reaction* OR life threatening allerg* OR allergic emergenc* OR anaphylactic reaction* | **Expanders** - Apply equivalent subjects | [306](javascript:__doPostBack('ctl00$ctl00$FindField$FindField$historyControl$HistoryRepeater$ctl08$linkResults','')) |
|  |  | **Search modes** - Proximity |  |
| S5 | anaphylaxis | **Expanders** - Apply equivalent subjects | [234](javascript:__doPostBack('ctl00$ctl00$FindField$FindField$historyControl$HistoryRepeater$ctl09$linkResults','')) |
|  |  | **Search modes** - Find all my search terms |  |
| S4 | S31 OR S32 OR S33 | **Expanders** - Apply equivalent subjects | [284,470](javascript:__doPostBack('ctl00$ctl00$FindField$FindField$historyControl$HistoryRepeater$ctl10$linkResults','')) |
|  |  | **Search modes** - Find all my search terms |  |
| S3 | quality of life OR wellbeing OR well-being OR health related quality of life OR QOL* OR HRQOL* | **Expanders** - Apply equivalent subjects | [262,145](javascript:__doPostBack('ctl00$ctl00$FindField$FindField$historyControl$HistoryRepeater$ctl11$linkResults','')) |
|  |  | **Search modes** - Proximity |  |
| S2 | quality N3 life | **Expanders** - Apply equivalent subjects | [121,551](javascript:__doPostBack('ctl00$ctl00$FindField$FindField$historyControl$HistoryRepeater$ctl12$linkResults','')) |
|  |  | **Search modes** - Find all my search terms |  |
| S1 | quality of life OR psychological well-being | **Expanders** - Apply equivalent subjects | [221,835](javascript:__doPostBack('ctl00$ctl00$FindField$FindField$historyControl$HistoryRepeater$ctl13$linkResults','')) |
|  |  | **Search modes** - Find all my search terms |  |

Embase

| Search | Term used | Result |
| --- | --- | --- |
| 19 | 6 and 10 and 18 included 2011 date parameter | 83 |
| 18 | 11 or 12 or 13 or 14 or 15 or 16 or 17 | 121013 |
| 17 | (psychological adj3 distress).af. | 41977 |
| 16 | (psychological adj3 state).af. | 6665 |
| 15 | (psychological adj3 well-being).af. | 47881 |
| 14 | (psychological adj3 well being).af. | 47881 |
| 13 | (psychological adj3 wellbeing).af. | 5835 |
| 12 | ("psychological well-being" or "psychological state" or "Psychological distress" or "Psychological resilience" or "Psychological stress").af. | 113396 |
| 11 | "psychological wellbeing".af. | 5080 |
| 10 | 7 or 8 or 9 | 111179 |
| 9 | "allergic disease*".af. | 41211 |
| 8 | ("Anaphylaxis" or "Allergic shock" or "Severe allergic reaction*" or "life threatening allerg*" or "Allergic emergenc*" or "Anaphylactic reaction*").af. | 73093 |
| 7 | "Anaphylaxis".af. | 70186 |
| 6 | 1 or 2 or 3 or 4 or 5 | 1125781 |
| 5 | "HRQOL*".af. | 41228 |
| 4 | "QOL*".af. | 111552 |
| 3 | ("Quality of life" or "wellbeing" or "well-being" or "health related quality of life").af. | 1104331 |
| 2 | (Quality adj3 life).af. | 905282 |
| 1 | ("Quality of life" or "Psychological well-being").af. | 923275 |

Cochrane Library

| **Search number** | **Search terms** | **Search options** | **Search number results Sep 02** |
| --- | --- | --- | --- |
| 1 | Psychological well-being | All | 9806 |
| 2 | Quality of life | All | 184697 |
| 3 | S1 or S2 | All | 190053 |
| 4 | (quality n3 life):ti,ab,kw | All | 130 |
| 5 | (quality of life):ti,ab,kw OR (wellbeing):ti,ab,kw OR (well-being):ti,ab,kw OR (health related quality of life):ti,ab,kw OR (QOL):ti,ab,kw | All | 198436 |
| 6 | (QOL*):ti,ab,kw OR (HRQOL*):ti,ab,kw | All | 37982 |
| 7 | #3 or #4 or #5 or #6 | All | 206305 |
| 8 | (Anaphylaxis):ti,ab,kw | All | 2063 |
| 9 | ((anaphylactic NEXT reaction*)):ti,ab,kw OR (allergic shock):ti,ab,kw OR (severe allergic reaction):ti,ab,kw OR (life threatening allergy):ti,ab,kw OR ((allergic NEXT emergency*)):ti,ab,kw | All | 1578 |
| 10 | allergic disease | All | 6533 |
| 11 | #8 or #9 or #10 | All | 9127 |
| 12 | psychological wellbeing | All | 876 |
| 13 | (psychological well-being):ti,ab,kw OR (psychological state):ti,ab,kw OR (psychological distress):ti,ab,kw OR (psychological resilience):ti,ab,kw OR (psychological stress):ti,ab,kw | All | 33312 |
| 14 | (psychological N3 (wellbeing or well being or well-being or wellness or stress* or resilience or state or distress)):ti,ab,kw | All | 8 |
| 15 | #12 or #13 or #14 | All | 33581 |
| 16 | #15 and #11 and #7 | All | 34 |
| 17 | #15 and #11 and #7 | Dates from 2011 | 22 |

Google Search- only exported the first 4 pages

| **Search number** | **Search terms** | **Search options** | **Search number results Sep 02** |
| --- | --- | --- | --- |
| 1 | Psychological well-being | All | 4,220,000 |
| 2 | Quality of life | All | 6,320,000 |
| 3 | S1 or S2 | All | 66,100 |
| 4 | (quality n3 life) | All | 2 |
| 5 | Quality of life OR "HRQOL*" OR "wellbeing" OR "well-being" OR "health related quality of life" OR "QOL* OR"quality n3 life" OR "Quality of life" | All | 34,900 |
| 6 | Anaphlaxis | All | 468,000 |
| 7 | "Anaphylaxis" OR "Anaphylactic reaction*" OR "Allergic shock" OR "Severe allergic reaction*" OR "life threatening allergy*" OR "Allergic emergenc*" OR "allergic disease*" | All | 348,000 |
| 8 | psychological wellbeing OR "psychological well-being" OR "Psychological stress" OR "psychological state" OR "Psychological distress" OR "Psychological resilience" OR "psychological N3 (wellbeing or well being or well-being or wellness or stress* or resilience or state or distress) " | All | 17800 |
| 9 | psychological wellbeing OR "psychological well-being" OR "Psychological stress" OR "psychological state" OR "Psychological distress" OR "Psychological resilience" OR "psychological N3 (wellbeing or well being or well-being or wellness or stress* or resilience or state or distress) " AND "Anaphylaxis" OR "Anaphylactic reaction*" OR "Allergic shock" OR "Severe allergic reaction*" OR "life threatening allergy*" OR "Allergic emergenc*" OR "allergic disease*" AND Quality of life OR "HRQOL*" OR "wellbeing" OR "well-being" OR "health related quality of life" OR "QOL* OR"quality n3 life" OR "Quality of life" | All | 1090 |
| 10 | psychological wellbeing OR "psychological well-being" OR "Psychological stress" OR "psychological state" OR "Psychological distress" OR "Psychological resilience" OR "psychological N3 (wellbeing or well being or well-being or wellness or stress* or resilience or state or distress) " AND "Anaphylaxis" OR "Anaphylactic reaction*" OR "Allergic shock" OR "Severe allergic reaction*" OR "life threatening allergy*" OR "Allergic emergenc*" OR "allergic disease*" AND Quality of life OR "HRQOL*" OR "wellbeing" OR "well-being" OR "health related quality of life" OR "QOL* OR"quality n3 life" OR "Quality of life" | Date range from 2011 | 17800 |

#### Appendix 4

Excluded studies and rational n=89

| Excluded original search = n=83 |  |  |
| --- | --- | --- |
| Authors | Published Year | Rational for exclusion |
| Al-Dairi, Walaa; Al Saeed, Ali A; Al Sowayigh, Omar M | 2020 | Exclusion reason: Not focused on anaphylaxis; |
| Anderson, Mary Alice | 2020 | Exclusion reason: Wrong study design; |
| Armisen, M: Guspi, R: Alfaya, T: Cruz, S: Fernandez, S: Dominquez-Noche, C: Alonso, A: Dalmau, G: Marques, L: Vega,A | 2015 | Exclusion reason: Wrong patient population; hand searched paper |
| Aqel S.; Imameldin A.O.; Ibrahim T. | 2023 | Exclusion reason: Wrong study design; |
| Åšwinoga, Magdalena; KÅ‚os, MaÅ‚gorzata; Miniszewska, Joanna; Zalewska-Janowska, Anna | 2012 | Exclusion reason: Wrong patient population; |
| Atiim, George A.; Elliott, Susan J.; Clarke, Ann E. | 2018 | Exclusion reason: Paediatric population; : population = allergy (not anaphylaxis) and anaphylaxis not reported separately that i can see; |
| Boyd H.; Gore C. | 2017 | Exclusion reason: Wrong patient population; |
| Bruin Weller, M. S.; Rockmann, H.; Knulst, A. C.; Bruijnzeelâ€Koomen, C. A. F. M. | 2013 | Exclusion reason: Wrong study design; |
| Budu-Aggrey A; Joyce S; Davies NM; Paternoster L; MunafÃ² MR; Brown SJ; Evans J; Sallis HM | 2021 | Exclusion reason: Not focused on anaphylaxis; |
| Burnell, Fiona J; Keijzers, Gerben; Smith, Pete | 2015 | Exclusion reason: Wrong study design; |
| Beyer, Susanne, Franke Annegret, Simon Jan Christoph, Treudler Regina | 2015 | Exclusion reason: Wrong patient population, from hand search |
| Chandravanshi, Jaipal; Kanwar, Ghanshyam Singh; Mishra, Vivekanand; Kumar, Ashutosh | 2024 | Exclusion reason: Wrong study design; |
| Childs, Caroline E; Munblit, Daniel; Ulfman, Laurien; GÃ³mez-Gallego, Carlos; Lehtoranta, Liisa; Recker, Tobias; Salminen, Seppo; Tiemessen, Machteld; Collado, Maria Carmen | 2022 | Exclusion reason: Wrong study design; |
| Cichocka, E: Brzyski, P: Swiebocka, E: Lange, J: Tobiasz, B: Liz, G: Jedynak-Waswicz, U: Kulus, M: Kaczmarski, M: Malaczyriska, T: Klajna-Kraluk: Breborrowicz, A: Kycler, Z: Pietrzyk, J. | 2012 | Exclusion reason: Wrong outcomes hand serached paper |
| Coelho G.L.D.H.; Byrne A.; Hourihane J.; DunnGalvin A. | 2021 | Exclusion reason: Wrong outcomes; |
| Das, Anupam; De, Abhishek; Godse, Kiran; Sangolli, Prabhakar; Zawar, Vijay; Sharma, Nidhi; Girdhar, Mukesh; Podder, Indrashis; Shah, Bela; Dhar, Sandipan | 2022 | Exclusion reason: Wrong study design; |
| Dias de castro, E: Barbosa,J: Mesquita, A: Caires, A: Ribeiro, L: Cernadas, J: Bairardina, I | 2021 | Exclusion reason: Wrong patient population (drug reaction only) |
| Dobler, Claudia C. | 2019 | Exclusion reason: Not focused on anaphylaxis; |
| Dorris, Stacy | 2020 | Exclusion reason: Wrong study design; |
| DunnGalvin, Audrey; Barnett, Julie; Begen, Fiona M; Ryan, Kathleen; Lucas, Jane S | 2018 | Exclusion reason: Wrong patient population |
| Durmaz K; Ataseven A; Temiz SA; Isik B; Dursun R | 2022 | Exclusion reason: Not focused on anaphylaxis; |
| Egmose, B: Huniche, L: Bindslev-Jensen, C: Nielsen, D: Mortz, C | 2024 | Exclusion reason: Wrong patient population |
| Fereidouni, Mohammad; Rezapour, Hadis; Saharkhiz, Mansoore; Mahmoudzadeh, Sara; Ayadilord, Malaksima; Askari, Masoumeh; Karbasi, Samira; Abbaszadeh, Arefeh; Hoseini, Zahra Sadat; Ferns, Gordon A.; Bahrami, Afsane | 2021 | Exclusion reason: Wrong patient population; |
| Ferrante, Giuliana; Montalbano, Laura; Cilluffo, Giovanna; Malizia, Velia; Marchese, Donatella; La Grutta, Stefania | 2016 | Exclusion reason: Not focused on anaphylaxis; |
| Ferreira, BÃ¡rbara Roque; Pio-Abreu, JosÃ© LuÃ­s; Figueiredo, AmÃ©rico; Misery, Laurent | 2021 | Exclusion reason: Wrong study design; |
| Fischer J; Feidt A; Giel KE; Martens U; Zipfel S; Biedermann T; Teufel M | 2011 | Exclusion reason: Wrong study design; hand searched item |
| Fukagawa, Kazumi; Fujishima, Hiroshi; Fukushima, Atsuki; Sumi, Tamaki; Okamoto, Shigeki; Shoji, Jun; Satake, Yoshiyuki; Ohno, Shigeaki; Namba, Kenichi; Kitaichi, Nobuyoshi; Ebihara, Nobuyuki; Takahashi, Hiroshi; Kumagai, Naoki; Uchino, Yuichi; Uchino, Miki; Murayama, Koichi; Sakata, Miki; Uchio, Eiichi; Takamura, Etsuko; Ohashi, Yuichi; Ohkubo, Kimihiro; Satoh, Toshihiko | 2012 | Exclusion reason: Not written in English; Full text not available in English. ; |
| GOODMAN, ROSE | 2023 | Exclusion reason: Wrong study design; |
| Gooseen, N: Flokstra-de Blok, B: Vlieg-Boerstra, B: Duiverman, E: Weiss, C: Furlong, T: Dubois, A | 2011 | Exclusion reason: Wrong patient population |
| Greenberger, Paul A.; Wallace, Dana V.; Lieberman, Phillip L.; Gregory, Sean M. | 2017 | Exclusion reason: Paediatric population; |
| Greenhawt, Matthew | 2016 | Exclusion reason: Wrong study design; |
| Greiwe, Justin | 2023 | Exclusion reason: Wrong study design; |
| Heisterberg MV; MennÃ© T; Johansen JD | 2014 | Exclusion reason: Not focused on anaphylaxis; |
| Herbert, Linda; Shemesh, Eyal; Bender, Bruce | 2016 | Exclusion reason: Paediatric population; |
| Hofer, V: Dolle-Bierke, S: Reinert, R: Worm, M | 2022 | Exclusion reason: Wrong Patient population |
| Hoyte, Flavia C L | 2017 | Exclusion reason: Wrong study design; |
| Ilaria B.; Fulvio B.; Omar F.; Rosaria C.; Walter C.G.; Antonino R. | 2011 | Exclusion reason: Wrong study design; |
| Jacobsen, Ryan C.; Gratton, Matthew C. | 2011 | Exclusion reason: Wrong outcomes; BETHANY ARBUCKLE (2025-02-27 15:41:54)(Select): Does not discuss QOL or PWB. ; |
| Jarosz M; Syed S; BÅ‚achut M; Badura Brzoza K | 2020 | Exclusion reason: Not focused on anaphylaxis; |
| Kansen HM; Le TM; Meijer Y; Flokstra-de Blok BMJ; Welsing PMJ; van der Ent CK; Knulst AC; van Erp FC | 2018 | Exclusion reason: Wrong study design; |
| Kawalec-Kajstura, Ewa; Chomicka, Magdalena; SuÅ‚kowska, Joanna; Puto, GraÅ¼yna; KuÅºmicz, Ilona | 2019 | Exclusion reason: Wrong patient population; |
| Kazufumi Yoshihara | 2015 | Exclusion reason: Wrong study design; |
| Kelman M; Hammersley V; Kendall M; Mukherjee M; Morrice L; Harley S; Schwarze J; Sheikh A | 2019 | Exclusion reason: Wrong study design; |
| Kerstin Hamberg Levedahl; Nilsson, Annika; Johansson, Birgitta; HedstrÃ¶m, Mariann | 2022 | Exclusion reason: Wrong patient population; |
| Knibb R; Halsey M; James P; du Toit G; Young J | 2019 | Exclusion reason: Wrong study design; |
| Knibb, Rebecca C.; Alviani, Cherry; Garrigaâ€Baraut, Teresa; Mortz, Charlotte G.; Vazquezâ€Ortiz, Marta; Angier, Elizabeth; Blumchen, Katerina; Comberiati, Pasquale; Duca, Bettina; DunnGalvin, Audrey; Gore, Claudia; Hox, Valerie; Jensen, Britt; Pite, Helena; Santos, Alexandra F.; Sanchezâ€Garcia, Silvia; Gowland, M. Hazel; Timmermans, Frans; Roberts, Graham | 2020 | Exclusion reason: Wrong study design; |
| Krasnick J; Patterson R; Meyers GL | 1996 | Exclusion reason: Wrong outcomes; |
| Lee, Youngsoo: Chang, Hyoung Yoon: Kim, Sang-Ha: Yang, Min-Suk: Koh, Young-Il: Kang, Hye-Ryun: Choi, Jeong-Hee: Kim, Cheol-Woo: Park, Hye-Kyung: Kim, Kim, Mi-ae: King, Jiwoong: Yoon, Jiwon: Ye, Young-Min | 2020 | Exclusion reason: Wrong outcomes; Paper from additional hand search |
| Lee, Gyu Na; Koo, Ha Yeh Rin; Han, Kyungdo; Lee, Young Bok | 2022 | Exclusion reason: Not focused on anaphylaxis; |
| Lind, Nina; Nordin, Maria; Palmquist, Eva; Claeson, Anna-Sara; Millqvist, Eva; Nordin, Steven | 2015 | Exclusion reason: Wrong outcomes; |
| Luebke, Carol Ann | 2017 | Exclusion reason: Wrong patient population; |
| Marginean, Cristina Maria; Popescu, Mihaela; Drocas, Andrei Ioan; Cazacu, Sergiu Marian; Mitrut, Radu; Marginean, Iulia Cristina; Iacob, George Alexandru; Popescu, Marian Sorin; Docea, Anca Oana; Mitrut, Paul | 2023 | Exclusion reason: Wrong study design; |
| Marshall, Gailen D; Tull, Matthew T | 2018 | Exclusion reason: Wrong study design; |
| Molzon ES; Bonner MS; Hullmann SE; Ramsey RR; Suorsa KI; Chaney JM; Mullins LL | 2013 | Exclusion reason: Not focused on anaphylaxis; |
| Muraro, A: Dubois, A: DunnGalvin, A: Hourinhane, O: Jong, W: Meyer, R: Panesar, S, Roberts, G: Salvilla, S: Sheikh, A: Wroth, A: Flokstra-de Blok, J | 2014 | Exclusion reason: Wrong outcomes hand searched paper |
| Nitin J; Palagani R; Shradha NH; Vaibhav J; Kowshik K; Manoharan R; Nelliyanil M | 2016 | Exclusion reason: Wrong outcomes; |
| Pandya, Samta P |  | Exclusion reason: Paediatric population; |
| Piontek K.; Arnold A.; Grabe H.; Volzke H.; Apfelbacher C. | 2022 | Exclusion reason: Wrong study design; |
| Polloni L; Muraro A | 2020 | Exclusion reason: Wrong study design; |
| Polloni L; Schiff S; Ferruzza E; Lazzarotto F; Bonaguro R; Toniolo A; Celegato N; Muraro A | 2017 | Exclusion reason: Wrong outcomes; |
| Polloni, L.; DunnGalvin, A.; Ferruzza, E.; Bonaguro, R.; Lazzarotto, F.; Toniolo, A.; Celegato, N.; Muraro, A. | 2017 | Exclusion reason: Paediatric population; |
| Pradhan, Sara | 2021 | Exclusion reason: Wrong study design; |
| Ramos, Ashley; Cooke, Frances; Miller, Emily; Herbert, Linda | 2021 | Exclusion reason: Wrong patient population; |
| Riaz, Ossama; Orimaye, Sylvester; Al-Khateeb, Thabit; Sodeke, Patrick; Awujoola, Adeola; Goodkin, Karl | 2020 | Exclusion reason: Wrong study design; |
| Roberts, Graham; Vazquezâ€Ortiz, Marta; Knibb, Rebecca; Khaleva, Ekaterina; Alviani, Cherry; Angier, Elizabeth; Blumchen, Katharina; Comberiati, Pasquale; Duca, Bettina; DunnGalvin, Audrey; Garrigaâ€Baraut, Teresa; Gore, Claudia; Gowland, M. Hazel; Hox, ValÃ©rie; Jensen, Britt; Mortz, Charlotte G.; Pfaar, Oliver; Pite, Helena; Santos, Alexandra F.; Sanchezâ€Garcia, Silvia | 2020 | Exclusion reason: Wrong study design; |
| Roberts, Kate; Meiser-Stedman, Richard; Brightwell, Alex; Young, Judith | 2021 | Exclusion reason: Paediatric population; |
| Rocheleau, Gregory C.; Rocheleau, Brandy N. | 2022 | Exclusion reason: Wrong patient population; |
| Rubeiz C.J.; Ernst M.M. | 2021 | Exclusion reason: Wrong study design; |
| Schoeben, L: Bubak, C: Schaarschmidt, M: Schmieder, A | 2020 | Exclusion reason: Same data study as Scaarschmidt et al., 2018 |
| Shin, Ji-Hyeon; Roh, Daeyoung; Lee, Dong-Hee; Kim, Soo Whan; Kim, Sung Won; Cho, Jin Hee; Kim, Byung-Guk; Kim, Boo-Young | 2018 | Exclusion reason: Not focused on anaphylaxis; |
| Shirkhani, Milad; Aali, Shahrbanoo; Khoshkhui, Maryam; Rafizadeh-Ardabili, Golnaz | 2022 | Exclusion reason: Wrong patient population; |
| Sigurgeirsdottir, Jonina; Halldorsdottir, Sigridur; Arnardottir, Ragnheidur Harpa; Gudmundsson, Gunnar; Bjornsson, Eythor Hreinn | 2019 | Exclusion reason: Wrong patient population; |
| Slade, Tim; Grove, Rachel; Burgess, Philip | 2011 | Exclusion reason: Wrong patient population; |
| Solley GO | 2004 | Exclusion reason: Wrong outcomes; |
| Sotomayor, Juan L. Jr. | 2014 | Exclusion reason: Wrong study design; |
| Spolakâ€Bobryk, Natalia; Romantowski, Jan; Kujawskaâ€Danecka, Hanna; Niedoszytko, Marek | 2022 | Exclusion reason: Wrong study design; |
| Tomljenovic D; Pinter D; Kalogjera L | 2014 | Exclusion reason: Not focused on anaphylaxis; |
| Tsoumani M; Regent L; Warner A; Gallop K; Patel R; Ryan R; Vereda A; Acaster S; DunnGalvin A; Byrne A | 2022 | Exclusion reason: Wrong patient population; |
| Vassilopoulou E; Skypala I; Feketea G; Gawlik R; Dunn Galvin A; Meyer R; Pitsios C; Maria Pop R; Ryan D; Said M; Schiere S; Vlieg-Boerstra B; Kull I | 2022 | Exclusion reason: Wrong study design; |
| Wang, Deping; Luo, Wenlong | 2016 | Exclusion reason: Wrong study design; |
| WOJCIECHOWSKA, MILENA; HNATYSZYN-DZIKOWSKA, ANNA; BECK, OLIWIA | 2014 | Exclusion reason: Wrong study design; |
| Yepes-NuÃ±ez, J J; GÃ³mez-GarcÃ­a, C; Espinosa-Herrera, Y; Cardona-Villa, R | 2012 | Exclusion reason: Wrong patient population; |
| Yonezawa, Kaori; Haruna, Megumi; Yamamoto-Hanada, Kiwako; Ohya, Yukihiro | 2022 | Exclusion reason: Wrong patient population; |
| Zellweger, F.; Eggel, A. | 2016 | Exclusion reason: Wrong study design; |
|  |  |  |
| Additional hand searched papers excluded n = 6 |  |  |
| Authors | Published Year | Rational for exclusion |
| Armisen, M: Guspi, R: Alfaya, T: Cruz, S: Fernandez, S: Dominquez-Noche, C: Alonso, A: Dalmau, G: Marques, L: Vega,A | 2015 | Exclusion reason: Wrong patient population; hand searched paper |
| Beyer, Susanne, Franke Annegret, Simon Jan Christoph, Treudler Regina | 2015 | Exclusion reason: Wrong patient population, from hand search |
| Cichocka, E: Brzyski, P: Swiebocka, E: Lange, J: Tobiasz, B: Liz, G: Jedynak-Waswicz, U: Kulus, M: Kaczmarski, M: Malaczyriska, T: Klajna-Kraluk: Breborrowicz, A: Kycler, Z: Pietrzyk, J. | 2012 | Exclusion reason: Wrong outcomes hand searched paper |
| Fischer J; Feidt A; Giel KE; Martens U; Zipfel S; Biedermann T; Teufel M | 2011 | Exclusion reason: Wrong study design; hand searched item |
| Lee, Youngsoo: Chang, Hyoung Yoon: Kim, Sang-Ha: Yang, Min-Suk: Koh, Young-Il: Kang, Hye-Ryun: Choi, Jeong-Hee: Kim, Cheol-Woo: Park, Hye-Kyung: Kim, Kim, Mi-ae: King, Jiwoong: Yoon, Jiwon: Ye, Young-Min | 2020 | Exclusion reason: Wrong outcomes; Paper from additional hand search |
| Muraro, A: Dubois, A: DunnGalvin, A: Hourinhane, O: Jong, W: Meyer, R: Panesar, S, Roberts, G: Salvilla, S: Sheikh, A: Wroth, A: Flokstra-de Blok, J | 2014 | Exclusion reason: Wrong outcomes hand searched paper |
